# Reference gene identification and validation for quantitative real-time PCR studies in developing *Xenopus laevis*

**DOI:** 10.1038/s41598-017-18684-1

**Published:** 2018-01-11

**Authors:** Bilal B. Mughal, Michelle Leemans, Petra Spirhanzlova, Barbara Demeneix, Jean-Baptiste Fini

**Affiliations:** CNRS/UMR7221, Muséum national d’Histoire naturelle/Université Paris-Sorbonne, Paris, France

## Abstract

Reference genes are essential for gene expression analysis when using real-time quantitative PCR (RT-qPCR). *Xenopus laevis* is a popular amphibian model for studying vertebrate embryogenesis and development. Further, *X. laevis* is ideal for studying thyroid signaling due to its thyroid dependent metamorphosis, a stage comparable to birth in humans. When using PCR based studies, a primary concern is the choice of reference genes. Commonly used references are *eef1a1*, *odc1*, *rpl8*, and *actnB*, although there is a lack of *ad hoc* reference genes for *X. laevis*. Here, we used previously published RNA-seq data on different *X. laevis* stages and identified the top 14 candidate genes with respect to their expression levels as a function of developmental stage and degree of variation. We further evaluated the stability of these and other candidate genes using RT-qPCR on various stages including the unfertilised eggs, whole embryos during early development and brains during late development. We used four different statistical software packages: deltaCT, geNorm, NormFinder and BestKeeper. We report optimized reference gene pair combinations for studying development (early whole embryos), brains at later stages (metamorphosis and adult), and  thyroid signalling. These reference gene pairs are suitable for studying different aspects of *X. laevis* development and organogenesis.

## Introduction

Several features make the African clawed frog, *Xenopus laevis*, an outstanding tool in biomedical research and vertebrate development. Since the 1930s, they have been used by doctors as a simple test for pregnancy. Egg production in female frogs is stimulated by the chorionic gonadotropin hormone found in pregnant woman’s urine. Biologists have utilized this same method to induce female frogs to lay eggs and then fertilise *in vitro* for synchronised development^1^. In turn, the synchronised development has been indispensable for developmental biologists to study regulatory and interactions networks that direct embryogenesis. The embryos develop externally and are free from direct maternal influences. This allows for embryos to be easily manipulated *e.g*. microinjections for genetic knockouts and knockdowns, germ layer dissections, tissue transplantations, etc. Rapid embryo growth means that a tadpole has fully functional set of organs within a couple of days. Coupled with a high number of brood size, *X. laevis* allows for large-scale genetic and chemical screens. Finally, the *X. laevis* genome has been fully sequenced and showed a remarkable structural similarity with the human genome^2,3^.

Real time quantitative polymerase chain reaction (RT-qPCR) is a widely popular technique for relative gene expression analysis, particularly during development. Reference genes within a RT-qPCR serve as internal controls for standard correction or normalisation^4^. Ideal reference genes should be stable throughout the diverse experimental conditions. Their expression is expected to be under different regulatory mechanisms/pathways than the ones being investigated. However, no single reference genes have been shown to be universally constant during development. Furthermore, many studies have shown that traditionally used popular housekeeping genes used to normalize gene expression vary considerably under different conditions^5–8^. There is currently only one study reporting the expression of reference genes for *X. laevis* ^7^ and another for its close relative, *Xenopus tropicalis* ^8^. In both cases, the reference genes characterised were the top most commonly used orthologues of genes found stably expressed in mammal tissues or were identified by systematic comparisons with traditional reference genes in mammals. These include the genes, glyceraldehyde-3-phosphate dehydrogenase (*gadph*), beta-actin (*actb*), H4-histone protein (*h4*), elongation factor eEF-1 alpha (*eef1a1*), and ornithine decarboxylase (*odc1*). However, several reports demonstrate that these choices can lead to inadequate normalization of gene expression. *gadph*, *actb* and *h4* have been shown to be differentially expressed during the pre- and post-natal periods in mammals^9–11^. The latter two, *eef1a1* and *odc1,* also exhibit varying expression levels before and after the mid-blastula (MBT) stage (shown below). There is, therefore, a need for identification and validation of alternative reference genes.

Deep RNA sequencing (RNA-seq) has become a powerful tool in high-throughput transcriptomic studies with its high resolution, large data set, and sensitivity. Recently, this  technique has been used to identify novel reference genes for model systems such as the human cell cultures, zebrafish, mice and plants^12–15^. In this study, we used the recent RNA-seq data from Session *et al*., to identify the most stably expressed genes during *X. laevis* development^3^. Their expression stability was further validated using RT-qPCR. We used four statistical algorithms, delta-CT^16^, geNorm^17^, BestKeeper^18^ and NormFinder^19^. According to their stability, we identified the best reference genes candidates during different stages of *X. laevis* development. We further identified and validated the reference gene candidates for stage NF48 *X. laevis* brains when studying thyroid hormone signalling and its disruption.

## Material and Methods

### Animal husbandry and collection

The *Xenopus laevis* strains (outbred, wildtype, Centre de Ressources Biologiques Xénopes Rennes) were maintained in accordance with institutional and European guidelines (2010/63/UE Directive 2010). All procedures and experiments performed were approved and in accordance by the local ethic committee (Museum National d’Histoire Naturelle, Project approval N°68.039). *X. laevis* eggs were obtained from females after injection with 500–800 units of human chorionic gonadotrophin hormone, hCG (MSD Santé Animale, France). Tadpoles were obtained by *in vitro* fertilisation and “de-jellied” using 2% cysteine solution (pH 7.8) in Marc’s Modified Ringer’s (MMR, 0.1 M NaCl, 2.0 mM KCl, 1 mM MgSO4, 2 mM CaCl2, 5 mM HEPES (pH 7.8), 0.1 mM EDTA). The animals were reared in 0.1x MMR (pH7.4) at 23°C. The tadpoles were staged according to the *Xenopus* table of development and collected^20^.

Early stage embryos including unfertilised oocytes (NF0), NF1, NF10, NF21, NF24, NF37, NF41 and NF50 were anesthetized with 0.01% MS-222, rinsed with sterile water, placed in 1.5 ml Sorenson tubes containing 100 μl lysis buffer (provided in RNAqueous micro kit (Ambion)) 3 embryos per tube, flash frozen in liquid nitrogen and stored at −80 °C until RNA extraction. For brain collection at stages, NF41, NF50, NF54, NF56, NF57, NF61 and NF66, the tadpoles were anesthetised in 0.01% MS-222, rinsed with sterile water, and their brains dissected on ice under sterile RNase-free conditions. A pool of 3 brains were placed in 1.5 ml Sorenson tubes containing 100 μl lysis buffer (provided in RNAqueous micro kit (Ambion)), flash frozen in liquid nitrogen and stored at −80 °C until RNA extraction. For each developmental stage, three biological replicates were collected.

Screening for thyroid signalling alterations with T_3_ (Sigma-Aldrich, Saint-Quentin Fallavier, France), NH3 (T_3_ antagonist synthesized by AGV Discovery (France)^21^ or Triclosan (Sigma-Aldrich), was carried out as previously described^22^. In summary, fifteen tadpoles were placed per well in 6 well plates (TPP Switzerland), containing either control solvent (DMSO) or chemical (T_3_, NH3 or Triclosan). DMSO concentration was 0.01% in all treatments. Plates were placed at 23 °C for 3 days. The water (and solutions) were renewed every day, at regular 24 h intervals. At 72 hours, brains were dissected for RNA extraction as described above.

### RNA extraction and cDNA synthesis

The RNA was extracted using the RNAqueous™-Micro Total RNA Isolation Kit (AMBION, ThermoFischer) using manufacturer’s instructions. In summary, the collected embryos were homogenised for 1 min at 30 Hz with a tissue disruptor, TissueLyser II (Qiagen, Netherlands). Lysis solution (provided in RNA extraction kit) was added to homogenised mix and vortexed. Samples were centrifuged at 12,000 g for 10 min at 4°C. The sample/lysate was passed through the Micro Filter Cartridge (RNA extraction kit), washed 3 times and eluted with 10 µl of pre heated elution solution. 1/10 of 10x DNase 1 buffer and 1 µl of Dnase 1 (provided in RNA extraction kit) was added to RNA elution and incubated for 20 mins at 37°C. 1/10 volume of DNase inactivation reagent (RNA extraction kit) was added and incubated for 2 mins at room temperature. The RNA elution was centrifuged for 90 secs at max speed to pellet the DNase Inactivation reagent, RNA transferred to RNase-free tubes and stored at −80°C until use. RNA concentrations were determined using a spectrophotometer (NanoDrop ThermoScientific, Rockford, IL) and RNA quality verified using BioAnalyzer (Agilent) where we only used samples with RNA Integrity Number (RIN) >7.5.

500 ng of RNA was used for reverse transcription using a High Capacity cDNA RT kit (Applied BioSystem, Foster City, CA) using manufacturer’s instructions in a total 20 µl reaction. The random primers used ensure that the first strand synthesis includes total RNA, including mRNA and rRNA. The cDNAs were stored at −20°C until use.

### Primer design

RT-qPCR primers for the novel candidate reference genes were designed using Primer3plus^23^ (Supplementary table 1). Primers for *eef1a1.S, odc1.L, dio1, dio2, dio3, thrα (tr-alpha)*, and *thr*β (*tr-beta)* were used from previously published data^22,24^. The primers were designed to target the exon-exon region where possible to avoid genomic DNA amplification. The specificity of each primer set was checked used Xenbase Blast^25^ and by manual alignment of the small and large copy of both genes. Transcript information of genes was based on *Xenopus laevis* 9.1 genome primary transcripts by JGI. Primers were ordered from Eurofins, Germany. Melting curves were analysed to ensure amplification specificity and null primer-dimer formation. Primer PCR efficiency was evaluated using serial dilutions of cDNA sample (1:10, 1:100, 1;1,000 & 1:10,000) in Expression Suite Software (Applied Biosystem). The amplification efficiency (E) of the primers are; *clta.L* (93.599%), *cfl1.S* (102.668%), *mlf2.S* (104.01%), *tm9sf4.L* (93.713%), *lpcat3.S* (121.422%), *slc35b1.L* (97.972%), *mtch2.L* (100.694%), *ube2m.S* (91.468%), *ppp1ca.L* (92.59%), *sub1.L* (97.972%), *mcts1.L* (116.719%), *ralb.S* (107.707%), *cox7b.S* (96.615%), and *prcp.S* (95.233%). The correlation coefficients (R^2^) of the standard curve varied from 0.932 to 0.996. For full primers sequences, please refer to Supplementary Table 1.

### RT-qPCR and data analysis

Real time quantitative PCR (RT-qPCR) was carried out using QuantStudio 6 flex (Life technologies) on 384 well-plates, with a standard reaction per well containing 1/20 diluted cDNA as template (1 μl per well) plus 5 μl of mix (Power SyBR mix, Applied BioSystem). The RT-qPCR reaction for each sample conducted in duplicates (technical replicate). Water and no-template controls were used as negative controls for each primer set.

The RT-qPCR data were analysed using the QuantStudio™ 6 and 7 Flex Real-Time PCR System (Life technologies). Cycle threshold (Ct) values were obtained using auto baseline and applied to all amplicons of the same primer set. Duplicates with the Ct value different of more than 0.5 were not considered and removed from analysis. qBase+ 2.6 was used for geNorm analysis. BestKeeper, NormFinder and delta-CT values were calculated using respective excel sheets provided by the authors^16–18^.

### Data analysis

**Figure 1:**
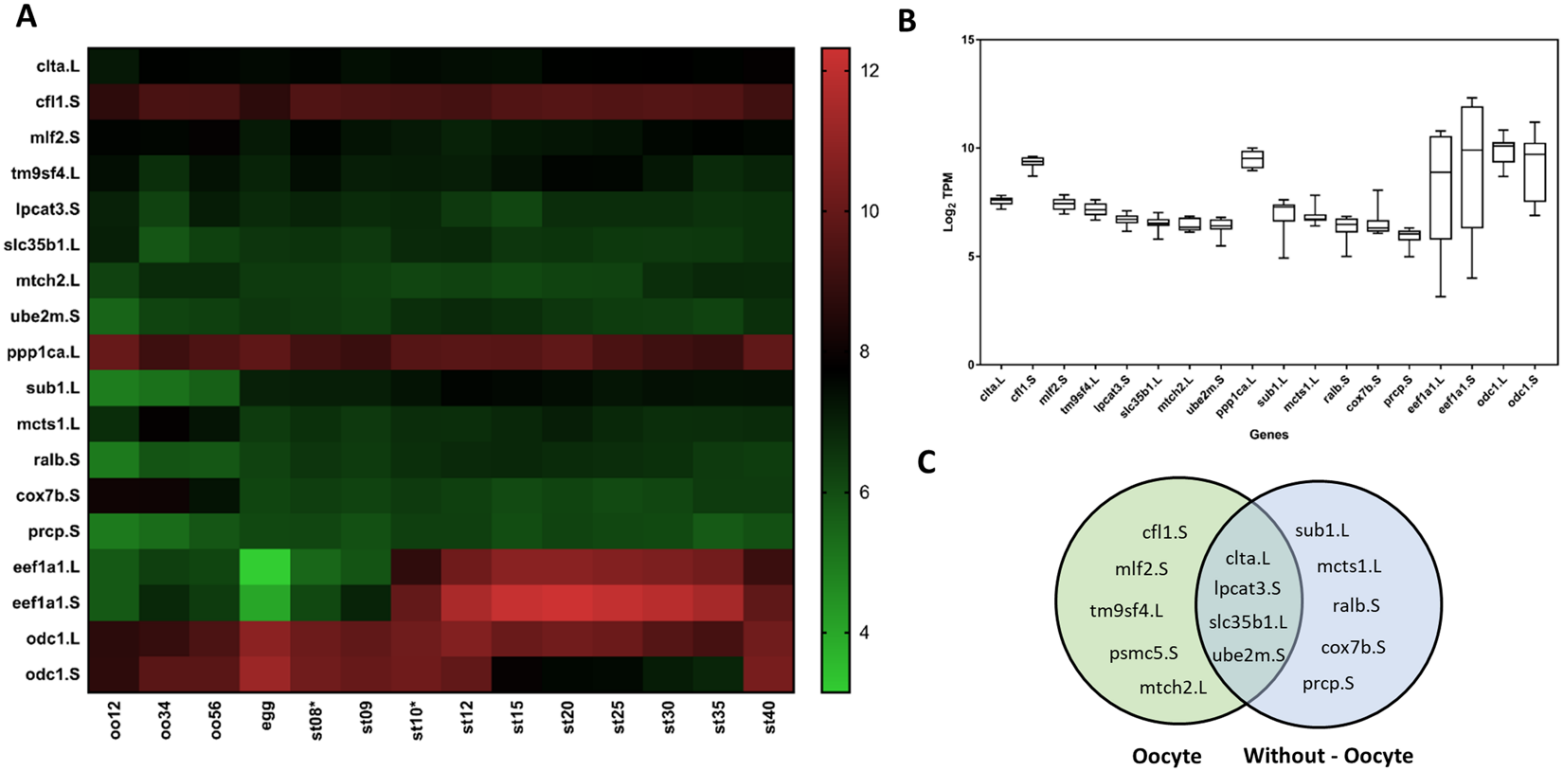
RNA-seq expression of 14 candidate reference genes and four previously used reference genes. (**A**) Heatmap of 18 candidate genes’ total RNA expression (TPM, transcript per million) during the 14 developmental stages of *Xenopus laevis* development. (**B**) Variation of 18 candidate genes’ total RNA expression (TPM) at the different stages as a box plot. (**C**) Venn diagram outlining the two different classes of top ranking genes, with oocyte and without oocyte.

The RNA-seq data set was obtained from Sessions *et al*., 2016^3^. Average TPM (transcript per million) value of the 14 developmental stages of individual gene was calculated. Standard deviation (SD) between the 14 developmental stages of each individual genes was then calculated. The genes were then ranked from least deviation, among the 14 developmental stages, to highest. Only genes which had an average value of more than 50 TPM were selected (see Supplementary Dataset 1 & 2, and Fig. 1). The Heatmap, box plots, and line graphs were generated using GraphPad Prism 7.0. Relative concentrations of cDNA for analysing relative changes in gene expression were calculated using 2^−ΔΔCt^ method^32^.

## Results

### Identification of candidate reference genes from *Xenopus laevis* RNA-seq data

In this study, RNA-seq data from 14 developmental *X. laevis* stages (including the unfertilised oocyte) by Session *et al*., was used to search and sort for candidate reference genes (45,831 genes)^3^. According to their paper, total RNA was extracted using Isogen (Nippon Gene) and the cDNA libraries were constructed using Illumina Truseq RNA sample prep kit V2 (Illumina), with the standard non-strand specific mRNA library preparation protocol. Our first step was to divide the RNA-seq data-set into two categories, with and without the oocyte stage (Fig. 1C). The genes in the two data sets were ranked and sorted according to two main criteria. First, a minimum mean expression across the stages was defined to be no lower than 50 TPM (transcripts per millions of read). Second, the genes were ranked according the least variation in expression in TPM among the different stages (see material and methods). Significant different rankings were observed for top genes for the two different data-sets (Table 1 and Supplementary Figure 1). We then selected the top 14 overlapping candidate genes for comparative expression analysis (Table 1 and Fig. 1). Note, *X. laevis* is an allotetraploid and contains two copies of certain genes (large (L) and small (S) chromosomes. We therefore differentiated and selected for one copy of the gene according to its ranking.

**Table 1:** Table of the 14 candidate reference genes and four previously used reference genes.

Gene	Name	Rank (Without Oocyte)	Rank (Oocytes)	Function
*clta.L*	Clathrin light chain A	1	1	Main structural component of the lattice-type cytoplasmic face of coated pits and vesicles which entrap specific macromolecules during receptor-mediated endocytosis
*sub1.L*	SUB1 Homolog, Transcriptional Regulator	2	588	General coactivator that functions cooperatively with TAFs and mediates functional interactions between upstream activators and the general transcriptional machinery
*mcts1.L*	Malignant T-Cell Amplified Sequence 1	3	135	Anti-oncogene that plays a role in cell cycle regulation; decreases cell doubling time and anchorage-dependent growth; shortens the duration of G1 transit time and G1/S transition
*lpcat3.S*	Lysophosphatidylcholine Acyltransferase 3	4	7	Involved in the pathway phospholipid metabolism, which is part of Lipid metabolism
*ralb.S*	RAS Like Proto-Oncogene B	5	28682	GTP-binding protein that belongs to the small GTPase superfamily and Ras family of proteins
*slc35b1.L*	Solute Carrier Family 35 Member B1	6	8	A nucleotide sugar transporter
*ube2m.S*	Ubiquitin Conjugating Enzyme E2 M	7	10	Encodes a member of the E2 ubiquitin-conjugating enzyme family
*cox7b.S*	Cytochrome C Oxidase Subunit 7B	8	3881	Terminal component of the mitochondrial respiratory chain, catalyzes the electron transfer from reduced cytochrome c to oxygen
*prcp.S*	Prolylcarboxypeptidase	9	44	Member of the peptidase S28 family of serine exopeptidases. An activator of the cell matrix-associated prekallikrein
*cfl1.S*	Cofilin 1	19	3	Polymerize and depolymerize F-actin and G-actin in a pH-dependent manner. Involved in the translocation of actin-cofilin complex from cytoplasm to nucleus.
*mlf2.S*	Myeloid Leukemia Factor 2	21	4	Unclear
*tm9sf4.L*	Transmembrane 9 Superfamily Member 4	49	5	Associates with proteins harbouring glycine-rich transmembrane domains and ensures their efficient localization to the cell surface
*mtch2.L*	Mitochondrial Carrier 2	58	9	Nuclear-encoded transporter, localized in the inner mitochondrial membrane. Thought to play a regulatory role in adipocyte differentiation and biology
*odc1.L*	Ornithine Decarboxylase 1	241	371	Rate-limiting enzyme of the polyamine biosynthesis pathway which catalyzes ornithine to putrescine
*eef1a1.S*	Eukaryotic Translation Elongation Factor 1 Alpha 1	3833	2580	Responsible for the enzymatic delivery of aminoacyl tRNAs to the ribosome
*eef1a1.L*	Eukaryotic Translation Elongation Factor 1 Alpha 1	3911	2690	Responsible for the enzymatic delivery of aminoacyl tRNAs to the ribosome
*odc1.S*	Ornithine Decarboxylase 1	3925	2677	Rate-limiting enzyme of the polyamine biosynthesis pathway which catalyzes ornithine to putrescine

The rank of each candidate gene was calculated by the least variation of expression in transcript per million (TPM) during mean expression <50 TPM between the 14 developmental stages from RNA-seq data.

In addition, both copies of the two commonly used developmental reference genes *eef1a* and *odc1* were selected and ranked (Table 1 and Fig. 1). Compared to the top 14 candidate reference genes, *eef1a1* and *odc1* ranked much lower (<2000) compared to candidate genes in both data-sets (Table 1). Expression heatmap of the 14 candidate genes revealed stable and consistent expression throughout the stages (Fig. 1A,B). In comparison, *eef1a1* and *odc* showing varying expression during the pre-and post mid blastula stages (Fig. 1A,B).

### Expression profile of candidate reference genes

Different rankings were observed for the isoforms of the candidate reference genes i.e. large vs small chromosome (Supplementary Table 1). Due to this, primer design for RT-qPCR was limited to just one copy of the gene (see material and methods). The performance of each primer pair was tested using a pool of cDNA for different stages and organs from the total RNA (see material and methods).

**Figure 2:**
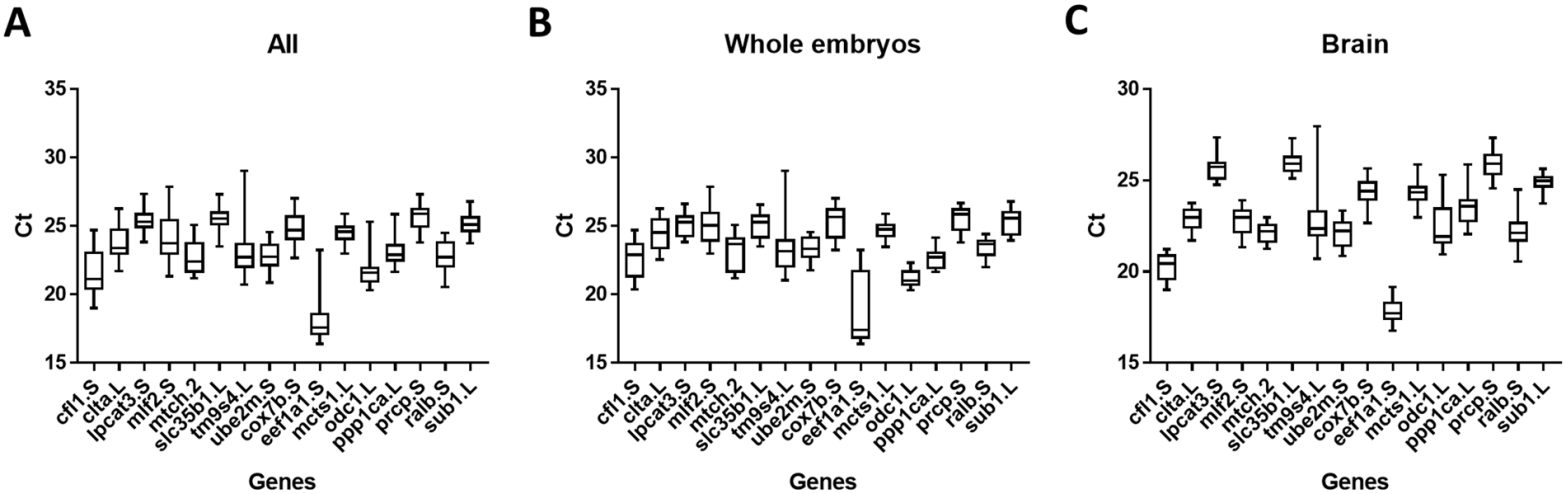
Ct values of 16 reference genes during different developmental stages. Variation of 16 candidate genes’ RNA expression (Ct) assessed using RT-qPCR in (**A**) whole embryos during early developmental stages and brain during metamorphic stages, (**B**) whole embryos during early developmental stages and in (**C**) brain during metamorphic stages.

The expression profiles candidate reference genes during the different stages of *X. laevis* development and brain were investigated. The expression levels of the candidate reference genes were determined by Ct (cycle threshold) values through RT-qPCR (Fig. 2). For each stage, the RT-qPCR was plotted with and without oocytes (Fig. 2 and Supplementary 2). For brain, the metamorphic stages from NF 41 onwards until Juvenile were used. Different ranges of the Ct values were found for all genes with varying standard deviations (Fig. 2 and Supplementary 2). In whole stages with oocytes, *odc1.L* had the least deviation (21.16 ± 0.5408) while *eef1a1.S* had the most (18.76 ± 2.596) (Supplementary Fig. 2A). In all stages without oocytes, *odc1.L* had the least deviation (21.08 ± 0.535) while *eef1a1.S* had the most (18.12 ± 2.085) (Supplementary Fig. 2A). In metamorphic brains, *sub1.L* had the least deviation (24.93 ± 0.4564) while *tm9sf4.L* had the most (22.64 ± 1.488) (Supplementary Fig. 2A).

### Expression stability of candidate reference genes

**Figure 3:**
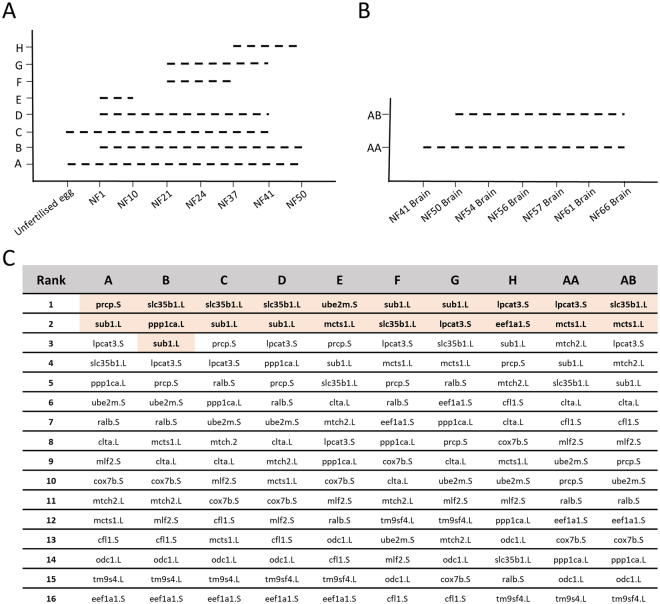
Stability of genes during different developmental periods. Gene expression and stability calculated using geNorm (qBase+). Different developmental series in (**A**) whole embryos during early developmental stages and (**B**) brain during metamorphic stages. The significance of the different developmental series are, A; whole embryo developmental stages including unfertilised egg (NF0–NF50), B; whole embryo developmental stages (NF1–NF50), C; whole embryo developmental stages prior to thyroid gland formation including unfertilised egg (NF0–NF41), D; whole embryo developmental stages prior to thyroid gland formation (NF1–NF41), E; whole embryo developmental stages from 1 cell to mid blastula (NF1–NF10), F; whole embryo developmental stages after gastrulation and prior to thyroid gland formation (NF21–NF37), G; whole embryo developmental stages after gastrulation and thyroid gland formation (NF21–NF41), H; whole embryo developmental stages during thyroid gland formation (NF37–NF50), AA, Brain tissue from early developmental period, metamorphosis and Juvenile (NF41–NF66), and AB; Brain tissue from metamorphosis and Juvenile stages (NF50–NF66). (**C**) Different series and their corresponding genes ranked using geNorm M. Highlighted genes are minimum combination of high ranking reference genes required in the series using geNorm V.

To evaluate stability of expression of the candidate reference genes, we initially used geNorm^17^. The geNorm algorithm is one of the most commonly used statistical analysis for ranking reference genes. It is based on the expression stability “M” value, derived from the average pairwise variation of a potential reference gene set with all other genes under investigation. Therefore, lower the M value, the higher the expression stability of the gene. We classified the various whole embryo and brain development stages into ten different developmental series (Fig. 3A and B). Series A encompasses stages from unfertilized eggs (NF0) until premetamorphic (NF50). Series B differs from Series A by exclusion of unfertilised eggs (NF0). Series C, D and G highlight developmental windows starting from unfertilised and fertilised eggs, and gastrulation (NF21) respectively and ending prior to thyroid gland formation (NF41). Series E focusses NF1 until gastrulation NF10 whereas series F and H are the stages adjacent to before and after the thyroid gland formation. Series AA and AB correspond to brain tissues during different developmental periods i.e. AA; Brain tissue from early developmental period, metamorphosis and Juvenile (NF41–NF66), and AB; Brain tissue from metamorphic and[H1] juvenile stages (NF50–NF66).

**Figure 4:**
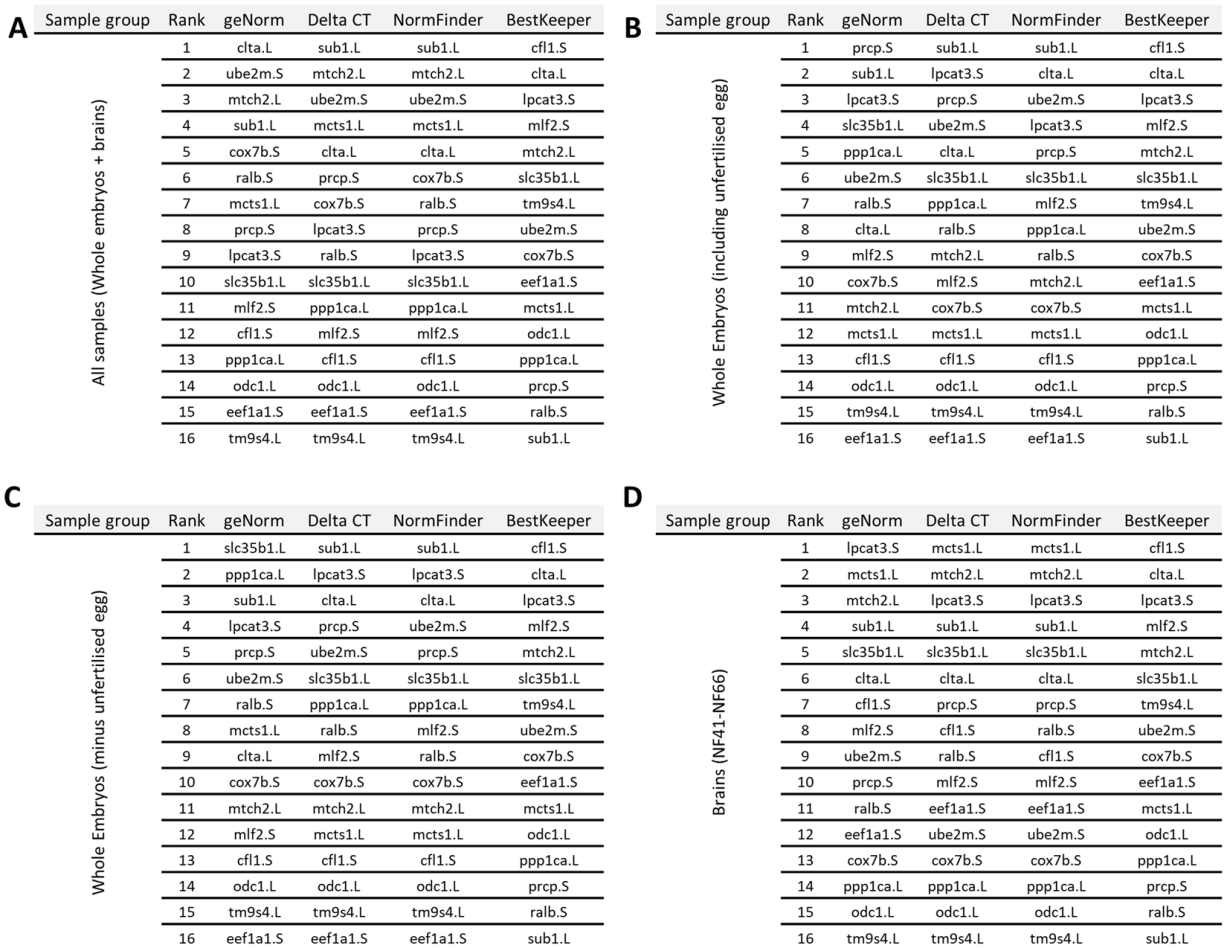
Comparison of four different statistical algorithms used to calculate reference gene stability. Four different statistical algorithms, geNorm, delta-CT, NormFinder and BestKeeper, were used to compare the gene expression and stability of the 16 candidate reference genes. Different developmental series (**A**) All samples including whole embryos and brain during metamorphosis, (**B**) whole embryos including unfertilised oocyte, (**C**) whole embryos without the unfertilised oocyte, and (**D**) brains during metamorphosis.

Using geNorm, we identified the top ranked candidate reference genes (Fig. 3C). Using the geNorm analysis of pairwise variation “V” value (V_n/n + 1_), we determined the optimal number of reference genes required for normalisation (Fig. 3 and Supplementary 3s). The genes *sub1.L* and *slc35b1.L*, were consistently the top ranked genes for early whole embryonic stages, while *lpcat1.S* and *sub1.L* were preferable for later stages. For brain during metamorphosis, *mcts1.L* and *slc35b1.L* were the top two minimum genes required. We further ranked and compared the candidate reference genes using delta-CT, BestKeeper and NormFinder with the geNorm (Fig. 4). delta-CT ranks candidate reference genes based on their delta-CT values where the genes with lowest delta-CT values have the most stable expression^16^. BestKeeper evaluates each candidate reference gene in terms of its coefficient of correlation to an index (geometric mean of all candidate genes). It also calculates both the SD and coefficient of variation (CV) of the original CT values. Candidate genes with the greatest stability possess the lowest calculated variations (CV ± SD). NormFinder is a model-based approach which ranks the candidate reference genes according to their expression stability^19^. The expression stability is calculated from linear scale expression transformed by delta-CT method where the lowest ranking represents lowest variation and most stable expression. We observed varying rankings for the top candidate genes with the four independent algorithms (Fig. 4 and Supplementary 4).

### Expression profile and stability of candidate reference genes compared to thyroid signalling components

We further evaluated the expression stability of the candidate reference genes in NF48 tadpole brains after a short 3 day exposure to thyroid hormone (T_3_), its antagonist (NH3)^21^ and known thyroid signalling altering chemical, Triclosan^26–28^. At NF48 stage, the tadpole thyroid gland is just beginning its formation and all the thyroid hormone action is carried out by the maternally available thyroid hormone^29^. Furthermore, this period of brain development is one of the most critical period for thyroid hormone action^22^. While the RNA-seq data did not contain any thyroid hormone exposed animals, we compared the identified candidate reference genes’ expression profile and stability with the commonly used housekeeping genes; *eef1a1.S* and *odc1.L*. In addition, we also compared the expression profile and stability of genes known to play an important role in thyroid hormone signalling pathway such as the deiodinases (*dio1, dio2* and *dio3*) and thyroid receptors alpha and beta (*trα* and *trβ*).

**Figure 5:**
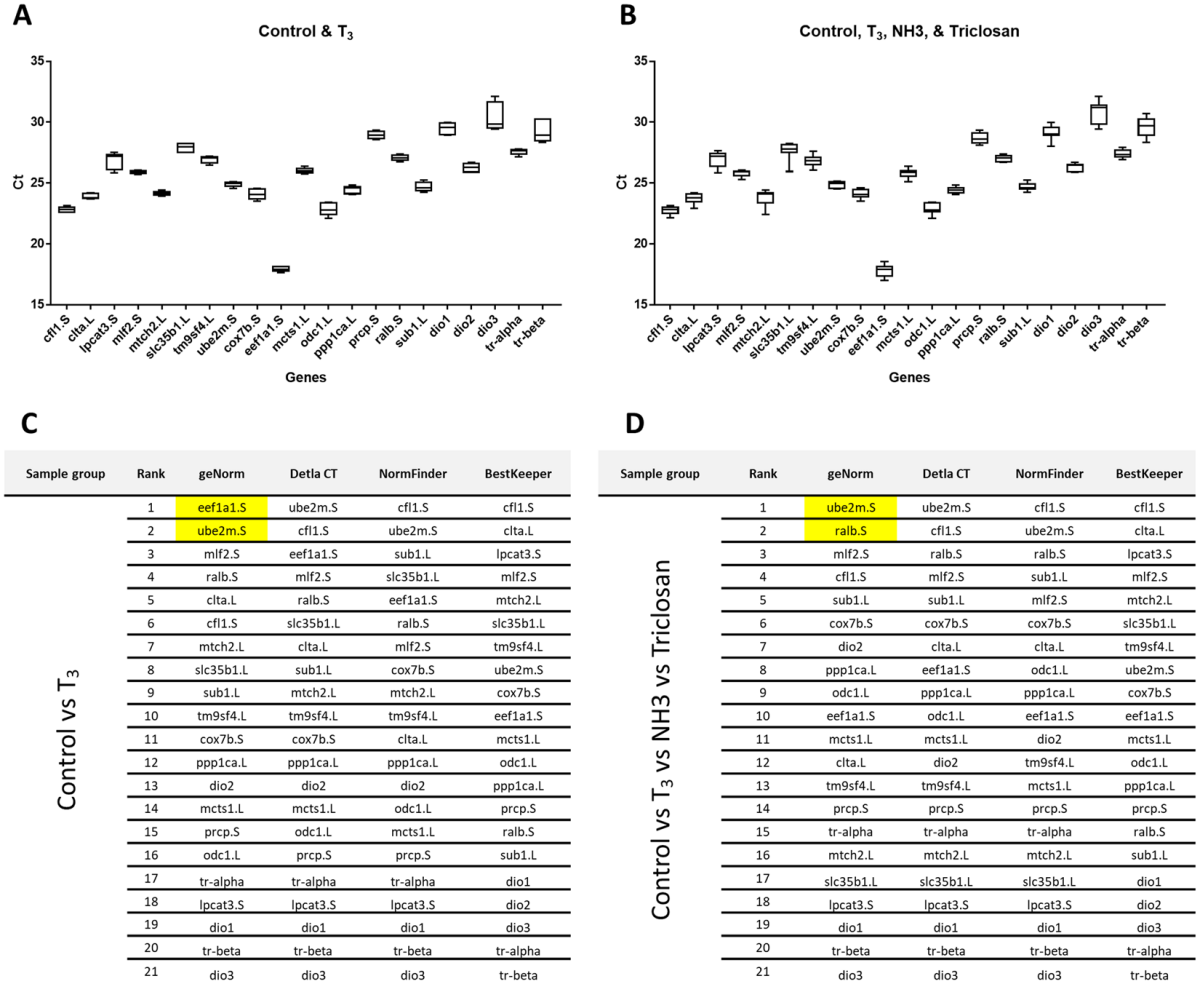
Ct values of 16 reference genes in NF48 brain exposed to thyroid (T_3_). Variation of 16 reference genes mRNA expression (Ct) assessed using RT-qPCR (**A**) Control vs T_3_ (**B**) Control vs T_3_ vs NH3 (T_3_ antagonist) vs Triclosan. Analysis using four different statistical algorithms, geNorm, delta-CT, NormFinder and BestKeeper. Series of different comparison of experimental conditions (**C**) Control vs T_3_ (**D**) Control vs T_3_ vs NH3 (T_3_ antagonist) vs Triclosan.

We split the experimental conditions into three main comparisons, i.e. Control vs T_3_, Control vs T_3_ vs NH3, and Control vs T_3_ vs NH3 vs Triclosan. As expected *dio3*, the inactivating enzyme of thyroid signalling, displayed the highest variation (Ct value) in all experimental conditions (30.44 ± 1.163, 30.75 ± 1.048 and 30.9 ± 0.9173 respectively) (Fig. 5 and Supplementary 5). The gene *mlf2.S* (Ct value) varied the least in Control vs T_3_ and the Control vs T_3_ vs NH3 exposed animals (25.9 ± 0.1287 and 25.8 ± 0.2377 respectively). Comparing controls to either T_3_, NH3 or Triclosan, *ppp1ca.L* found to vary the least (24.4 ± 0.243). geNorm algorithm ranked *eef1a1.S* and *ube2m.S* as the top ranked genes for Control vs T_3_ while *ube2m.S* and *ralb.S* were ranked highest for Control vs T_3_ vs NH3 vs Triclosan (Fig. 5C and Supplementary 5). Varying rankings were observed for the three other statistical programs in a similar fashion to the previous developmental stages.

**Figure 6:**
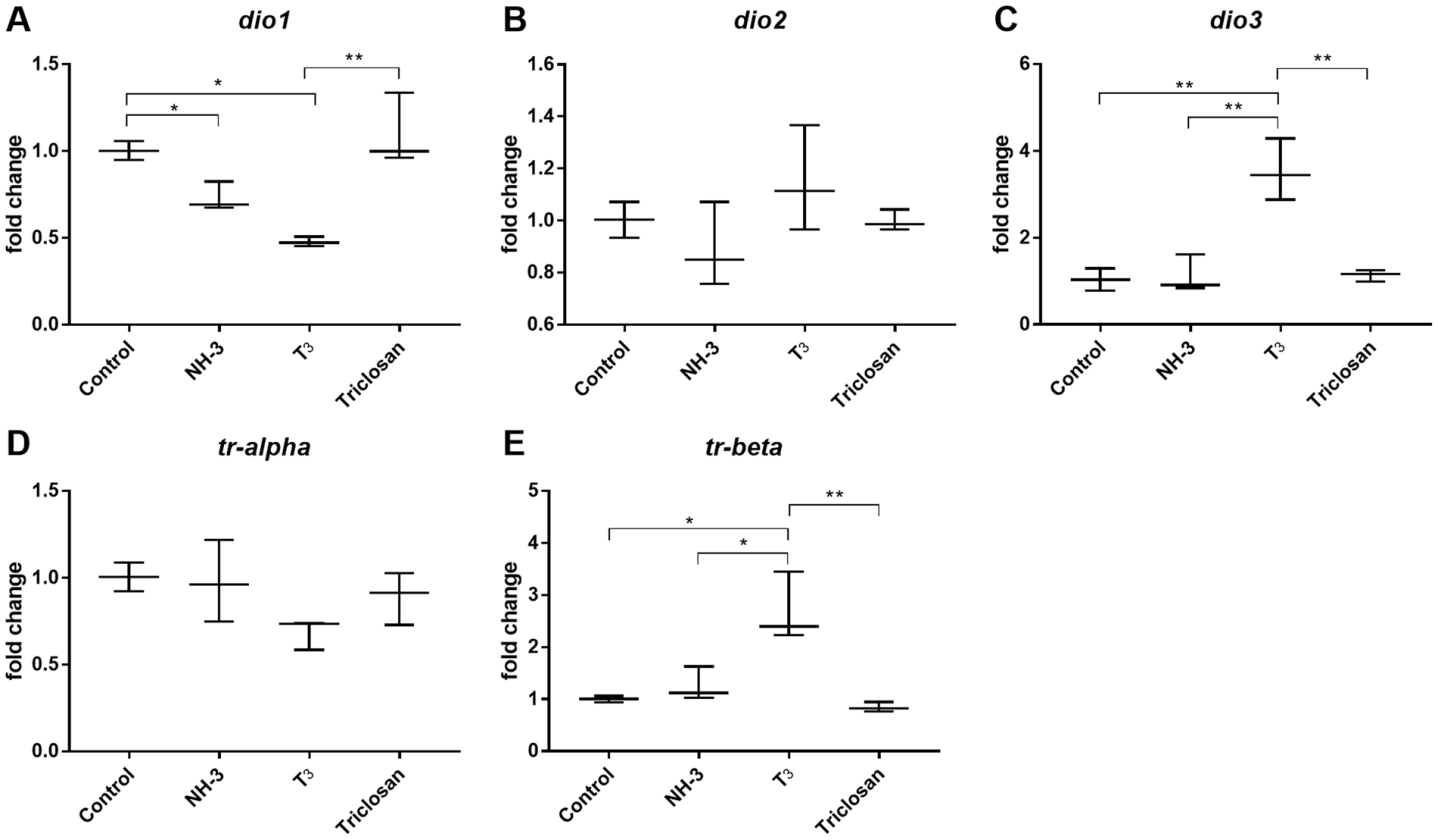
Relative fold changes of thyroid signalling genes using reference genes in NF48 *X. laevis* brains. Results are presented as fold changes. The previously identified two candidate reference genes, *ube2m.S* and *ralb.S*, were used to normalise the expression of the thyroid signalling genes (**A**) *dio1*, (**B**) *dio2*, (**C**) *dio3*, (**C**) *tr* *α*, and (**E**) *tr* *β*. Statistics used one way ANOVA. Values represent means ± SD (n = 3); *P < 0.01, and **P < 0.001.

Finally, we used the two candidate reference genes identified from the Control vs T_3_ vs NH3 vs Triclosan group (Fig. 5D), i.e. *ube2m.S* and *ralb.S*, as normalisers and calculated the relative fold change of thyroid signalling genes (Fig. 6). As expected, altered expression levels of *dio1*, *dio3* and *tr* *β* were observed under different experimental conditions (Fig. 6A,B and E respectively). No changes were observed for *dio2* which is not surprising since *dio2* is known to be regulated post translationally^30,31^. No change in gene expression was observed for Triclosan when compared to control. This may perhaps be due to the short exposure time (3 days) and low exposure concentration (10^−7^ M).

## Conclusion

We have identified and characterised suitable reference genes for studying development (early whole embryo stages from NF0 to NF50), brains at later stages (including metamorphosis and adult), and thyroid signalling in *X. laevis*. We focused on identifying novel reference genes from the previously published RNA-seq data of 45,831 genes. We report various combinations of reference genes including *sub1.L* and *slc35b1.L* for early whole embryonic stages, and *lpcat1.S* and *sub1.L*, for latter whole embryos, and *mcts1.L* and *slc35b1.L f*or brain during metamorphosis. These reference gene combinations will be of use to those studying early developmental networks during the different periods of embryogenesis. For NF48 brains exposed to thyroid hormone signalling and its antagonists/agonists, we report an optimal combination including *eef1a1.S*, *ube2m.S* and *ralb.S*. In addition, we further report the varying rankings by the four most common statistical algorithms used to characterise reference gene expression and stability. The differences in rankings are open to interpretation by the scientific community and their selection of reference genes according to their preferred statistical algorithm.

## Electronic supplementary material

Supplementary Information

Supplementary Dataset 1

Supplementary Dataset 1
