# Supplementary material for: Reference gene identification and validation for quantitative real-time PCR studies in developing Xenopus laevis
Source: Sci Rep. 2018 Jan 11;8:496. doi: 10.1038/s41598-017-18684-1 (PMC5764961; doi:10.1038/s41598-017-18684-1)
Supplement: Supplementary file 1 — Supplementary Information [file 41598_2017_18684_MOESM1_ESM.pdf]

Reference gene identification and validation for quantitative real-time PCR studies in developing *Xenopus laevis*

Bilal B Mughal, Michelle Leemans, Petra Spirhanzlova, Barbara Demeneix & Jean-Baptiste Fini

CNRS / UMR7221, Museum National d'Histoire Naturelle, Paris, France

Supplementary figures

Supplementary figure legends

- **Supplementary figure 1:** (Table) List of genes ranked according their high mean expression levels during (more than 50 TPM) and the least variation between the stages.

- **Supplementary figure 2: (A)** Ranking of genes' Ct variation across the different developmental stages in whole embryos during early developmental stages and in brain during metamorphic stages. **(B)** Variation of 16 candidate genes RNA expression during different individual stages (whole embryo) using RT-qPCR. **(C)** Variation of 16 candidate genes RNA expression during different individual stages (metamorphic brains) using RT-qPCR.

- **Supplementary figure 3**: Pairwise Variation (V) analysis using geNorm of 16 reference genes in whole embryos during early developmental stages and in brain during metamorphic stages.

- **Supplementary figure 4: Comparison of four different statistical algorithms used to calculate reference gene stability**. Four different statistical algorithms, geNorm, delta-CT, NormFinder and BestKeeper, were used to compare the gene expression and stability of the 16 candidate reference genes.

- **Supplementary figure 5: Ct values of 16 reference genes in NF48 brain exposed to thyroid (T_3).** Variation of 16 reference genes mRNA expression (Ct) assessed using RT-qPCR **(A)** Control vs T3 vs NH3 (T3 antagonist). **(B)** Variation of 16 candidate genes RNA expression during different series of experimental conditions Control vs T3, Control vs T3 vs NH3 (T3 antagonist) and, Control vs T3 vs NH3 (T3 antagonist) vs Triclosan. **(C)** Analysis using four different statistical algorithms, geNorm, delta-CT, NormFinder and BestKeeper on series of different experimental conditions. (D) Pairwise Variation (V) analysis using geNorm of 16 reference genes on series of different experimental conditions.

- **Supplementary Table 1:** Primers for gene expression (RT-qPCR). See material and methods

Supplementary figure 1

Attached separately as Supplementary Dataset

Screenshot of first page top 50 genes

1	GeneName	SeqID	oo12	oo34	oo56	egg	st08*	st09	st10*	st12	st15	st20	st25	st30	st35	st40	Mean	SD
2	clta.L	Xelaev18006549m	145.17	206	195.65	182.375	195.88	168.035	181.677	173.865	166.225	204.51	207.485	211.965	200.517	225.17	190.323143	21.6838999
3	ier3ip1.S	Xelaev18011117m	30.46	27.66	26.34	16.35	20.33	19.735	21.783	20.745	24.53	21.705	23.51	25.43	23.73	20.73	23.0741429	3.64683534
4	cfl1.S	Xelaev18024191m	423.03	658.41	643.24	418.055	724.29	675.475	639.967	610.595	738.195	785.005	742.98	770.815	742.8	570.55	653.1005	116.779706
5	mlf2.S	Xelaev18036719m	197.8	193.26	229.48	140.625	196.557	157.74	142.743	124.87	145.625	151.715	158.565	188.87	200.56	190.125	172.7525	30.3797137
6	tm9sf4.L	Xelaev18043054m	170.94	102.48	158.74	122.065	170.117	131.565	136.633	133.265	161.625	195.775	192.37	148.91	109.283	121.94	146.836286	29.2057323
7	psmc5.S	Xelaev18046105m	166.69	84.77	104.22	150.205	110.813	100.83	119.13	105.035	118.95	150.65	143.16	144.78	140.113	153.985	128.095071	24.7947061
8	lpcat3.S	Xelaev18036702m	125.87	75.68	137.57	110.71	123.407	108.68	114.423	87.015	71.425	104.52	105.305	104.345	96.97	100.425	104.738929	18.3255431
9	slc35b1.L	Xelaev18043267m	130.19	55.82	77.31	92.82	95.737	86.59	111.363	113.715	91.835	94.8	87.755	87.235	87.923	100.805	93.8498571	17.4718941
10	mtch2.L	Xelaev18021940m	75.4	108.83	108.3	85.34	85.31	77.77	72.15	76.245	69.64	74.46	76.06	102.795	115.763	113.45	88.6795	17.1127605
11	ube2m.S	Xelaev18037635m	44.9	73.96	77.97	91.645	86.563	81.17	103.353	111.485	103.025	90.995	83.575	81.625	75.273	101.71	86.2320714	16.6542135
12	unnamed	Xelaev18032016m	31.09	58.09	45.45	38.875	50.69	55.975	59.587	59.76	58.06	45.115	54.26	55.04	45.56	41.52	49.9337143	8.8351907
13	timm8b.S	Xelaev18037434m	18.84	32.37	25.83	24.08	20.273	26.24	23.113	15.8	26.115	21.965	26.03	23.9	16.43	19.07	22.8611429	4.49931542
14	fbxo9.L	Xelaev18027343m	10.09	17.45	17.88	12.305	11.787	12.92	11.7	15.13	18.91	15.435	19.725	15.255	14.447	13.195	14.7306429	2.9304864
15	phyhd1.S	Xelaev18041675m	12.07	11.48	11.14	12.995	11.6	10.285	11.373	11.015	9.64	9.025	8.955	10.235	13.173	17.52	11.4647143	2.16815904
16	LOC100496229.S	Xelaev18024241m	2.87	2.45	3.46	2.295	3.477	4.05	3.467	2.94	4.055	2.805	3.165	2.49	2.69	2.675	3.0635	0.56591597
17	ppp1ca.L	Xelaev18003383m	1022.43	541.73	695.04	910.57	590.06	514.78	785.24	829.895	805.975	925.93	662.325	553.045	498.633	939.935	733.970571	177.841408
18	ppp1ca.S	Xelaev18002199m	845.34	429.1	563.96	802.085	510.473	424.58	585.18	598.8	633.31	896.675	671.8	585.555	547.067	863.825	639.839286	155.716401
19	unnamed	Xelaev18042903m	382.9	324.77	299.8	341.65	229.873	211.445	293.867	349.895	402.61	439.755	329.26	250.115	189.15	316.055	311.510357	72.6441359
20	ssr3.L	Xelaev18027671m	198.42	263.49	221.68	365.85	239.153	233.015	244.677	320.935	368.85	330.355	283.56	253.37	306.857	436.655	290.4905	67.5801153
21	Xetrov90009588m.1	Xelaev18004378m	227.09	385.31	268.61	398.265	234.597	227.07	272.907	312.235	269.77	258.885	210.705	191.54	172.653	312.94	267.326929	66.503801
22	psmb1.S	Xelaev18029019m	189.87	360.14	386.19	188.695	226.727	232.075	228.707	204.745	207.66	279.9	294.19	320.505	337.883	262.47	265.696929	65.3138047
23	unnamed	Xelaev18028379m	162.63	325.11	302.64	237.73	227.88	197.955	257.817	322.345	327.26	319.15	250.83	211.93	180.513	229.04	253.773571	56.8118916
24	eif6.L	Xelaev18043248m	260.68	342.75	337.52	230.23	232.513	200.295	141.95	167.315	194.69	262.305	269.94	272.585	240.44	252.075	243.234857	56.5879004
25	unnamed	Xelaev18022097m	316.12	314.16	226.28	241.29	154.127	163.48	232.433	285.27	340.045	259.06	224.96	198.965	160.083	237.94	239.586643	59.1548907
26	uqcrfs1.S	Xelaev18024969m	233.18	213.54	246.89	371.265	227.567	194.11	193.37	210.41	141.2	227.73	211.865	222.69	209.69	330.415	230.994429	57.0400561
27	tax1bp3.S	Xelaev18014203m	300.64	162.97	232.65	214.335	200.623	196.57	221.447	203.01	135.78	202.105	164.035	139.665	125.227	172.895	190.853714	45.9676174
28	unnamed	Xelaev18024234m	244.41	224.05	159.97	207.37	117.487	133.87	134.32	134.145	135.83	185.29	188.56	183.27	140.143	232.05	172.911786	42.1735311
29	unnamed	Xelaev18036094m	163.66	257.99	231.18	102.055	181.037	178.055	213.38	170.12	122.11	145.355	152.57	167.885	161.163	131.55	169.865	42.0401223
30	unnamed	Xelaev18004428m	98.24	96.82	166.32	229.635	209.85	171.545	182.207	167.485	145.52	206.215	200.29	169.38	127.587	173.43	167.466	39.5842117
31	dcaf7.S	Xelaev18046098m	147.67	142.22	157.99	251.305	156.293	120.895	131.687	141.69	113.81	165.7	154.395	142.52	142.23	232.84	157.231786	38.7355474
32	brk1.S	Xelaev18025828m	137.99	206.52	131.18	215.925	126.977	123.45	158.237	160.385	119.965	123.11	117.765	135.88	146.07	227.52	152.212429	37.4880662
33	thoc3-like.L	Xelaev18017283m	105.9	109.82	153.39	80.85	149.307	142.175	148.13	185.76	159.385	176.145	147.145	116.325	97.78	87.94	132.860857	32.9055843
34	smarcb1.S	Xelaev18010510m	119.64	133.46	154.34	149.82	157.01	126.485	128.25	123.965	95.96	162.59	122.085	89.805	68.287	123.635	125.380857	26.7974741
35	unnamed	Xelaev18001841m	81.68	90.37	133.02	122.045	176.313	169.38	161.933	90.83	101.79	119.975	118.615	131.825	123.153	131.515	125.174571	29.0621205
36	unnamed	Xelaev18011472m	183.69	135.21	96.47	133.84	94.29	85.755	124.723	121.35	115.765	124.38	164.02	152.755	97.847	93.935	123.145	29.0493683
37	Xetrov90029291m.L	Xelaev18019582m	171.04	89.92	135.18	76.17	108.913	92.455	113.19	96.285	106.725	137.07	131.6	124.115	106.613	71.575	111.489357	26.7194495
38	txn2.S	Xelaev18025552m	102.54	89.59	109.23	92.79	100.15	85.325	59.873	60.705	75.645	84.61	86.39	101.985	124.927	130.545	93.1646429	20.6455085
39	unnamed	Xelaev18028531m	66.56	86.35	105.94	128.95	118.783	104.575	106.82	97.045	78.175	92.065	79.07	69.67	48.133	103.49	91.8304286	21.9652982
40	npc2.S	Xelaev18041377m	57.75	116.16	91.88	30.31	71.54	74.995	100.377	81.355	85.425	81.045	88.245	94.305	93.79	77.755	81.7808571	20.3440747
41	tm9sf4.S	Xelaev18045841m	107.21	59.06	82.36	98.85	104.75	79.84	75.867	58.295	71.36	81.865	82.445	67.41	52.547	79.68	78.6813571	16.6700485
42	mcm7.1	Xelaev18000877m	77.01	66.08	78.82	117.145	72.68	57.9	54.3	56.335	58.42	85.095	85.175	69.695	58.037	99.075	73.9833571	18.2090906
43	MGC89864.L	Xelaev18035078m	66.57	101.57	67.32	51.98	56.917	66.32	66.103	55.28	41.785	52.64	61.81	76.555	78.167	69.545	65.183	14.4287718
44	unnamed	Xelaev18019735m	58.92	34.68	69.36	48.2	75.07	60.63	67.99	79.88	65.335	79.865	75.705	71.04	58.79	45.105	63.6121429	13.5531513
45	prcp.S	Xelaev18016367m	31.85	39.31	55.54	69.16	70.927	60.73	79.587	79.87	64.435	74.375	70.095	67.85	52.413	60.795	62.6383571	14.069275
46	LOC100498237-like.L	Xelaev18043652m	50.31	54.22	38.16	53.185	67.05	60.71	81.123	80.515	79.11	65.97	48.845	51.205	41.403	73.145	60.3536429	14.3781214
47	tmed4.S	Xelaev18020264m	30.44	43.19	43.48	59.1	65.603	59.96	57.623	45.55	66.085	77.305	77.84	70.86	64.223	73.12	59.5985	14.2406371
48	unnamed	Xelaev18034134m	52.86	59.2	56.07	41.86	63.703	61.39	56.367	40.82	39.37	61.425	77.49	73.885	55.32	47.675	56.2453571	11.4206479
49	ppp5c.S	Xelaev18042357m	20.94	56.57	50.62	35.37	49.61	44.04	46.37	44.47	57.67	54.145	65.23	69.03	59.637	51.895	50.3997857	12.234347
50	unnamed	Xelaev18016649m	49.96	28.69	56.78	36.855	54.43	42.085	45.05	49.42	51.07	70.14	67.6	51.89	47.073	36.29	49.0952143	11.3853998
51	[illegible]	[illegible]	[illegible]	[illegible]	[illegible]	[illegible]	[illegible]	[illegible]	[illegible]	[illegible]	[illegible]	[illegible]	[illegible]	[illegible]	[illegible]	[illegible]	[illegible]	[illegible]

Supplementary figure 1: List of genes ranked according their high mean expression levels during (more than 50 TPM) and the least variation between the stages. Green = TPM>200, Yellow, TPM 50-200, Red = TPM<50.

Supplementary figure 2a

Page 1/3

Rank	Whole embryo + Oocyte			Whole embryo - Oocyte			Brain		
	Gene	Mean	Std Deviation	Gene	Mean	Std Deviation	Gene	Mean	Std Deviation
1	odc1.L	21.16	0.5408	odc1.L	21.08	0.535	sub1.L	24.93	0.4564
2	mcts1.L	24.74	0.592	mcts1.L	24.77	0.6279	mtch.2	22.13	0.5497
3	ppp1ca.L	22.61	0.6427	ppp1ca.L	22.55	0.6674	slc35b1.L	25.92	0.5684
4	ralb.S	23.45	0.7096	ralb.S	23.36	0.7183	eef1a1.S	17.83	0.5774
5	lpcat3.S	25.08	0.7778	lpcat3.S	25	0.7951	clta.L	22.94	0.5981
6	ube2m.S	23.4	0.817	ube2m.S	23.28	0.8066	lpcat3.S	25.65	0.6048
7	slc35b1.L	25.03	0.8563	sub1.L	25.18	0.8398	mcts1.L	24.28	0.6065
8	prcp.S	25.49	0.8603	slc35b1.L	24.92	0.8573	cox7b.S	24.44	0.6624
9	sub1.L	25.33	0.8794	prcp.S	25.37	0.8616	prcp.S	25.86	0.7009
10	clta.L	24.45	1.141	clta.L	24.27	1.106	cfl1.S	20.28	0.7162
11	cox7b.S	25.28	1.153	cox7b.S	25.22	1.226	ube2m.S	22.11	0.7231
12	mlf2.S	25.09	1.257	mlf2.S	24.95	1.28	mlf2.S	22.78	0.7612
13	mtch.2	23.15	1.277	mtch.2	23.02	1.311	ralb.S	22.19	0.8204
14	cfl1.S	22.61	1.405	cfl1.S	22.51	1.472	ppp1ca.L	23.54	0.9991
15	tm9s4.L	23.61	2.213	tm9s4.L	23.09	1.608	odc1.L	22.45	1.159
16	eef1a1.S	18.76	2.596	eef1a1.S	18.12	2.085	tm9s4.L	22.64	1.488

Supplementary figure 2: (A) Ranking of genes' Ct variation across the different developmental stages in whole embryos during early developmental stages and in brain during metamorphic stages. **(B)** Variation of 16 candidate genes RNA expression during different individual stages (whole embryo) using RT-qPCR. **(C)** Variation of 16 candidate genes RNA expression during different individual stages (metamorphic brains) using RT-qPCR.

Supplementary figure 2b

Page 2/3

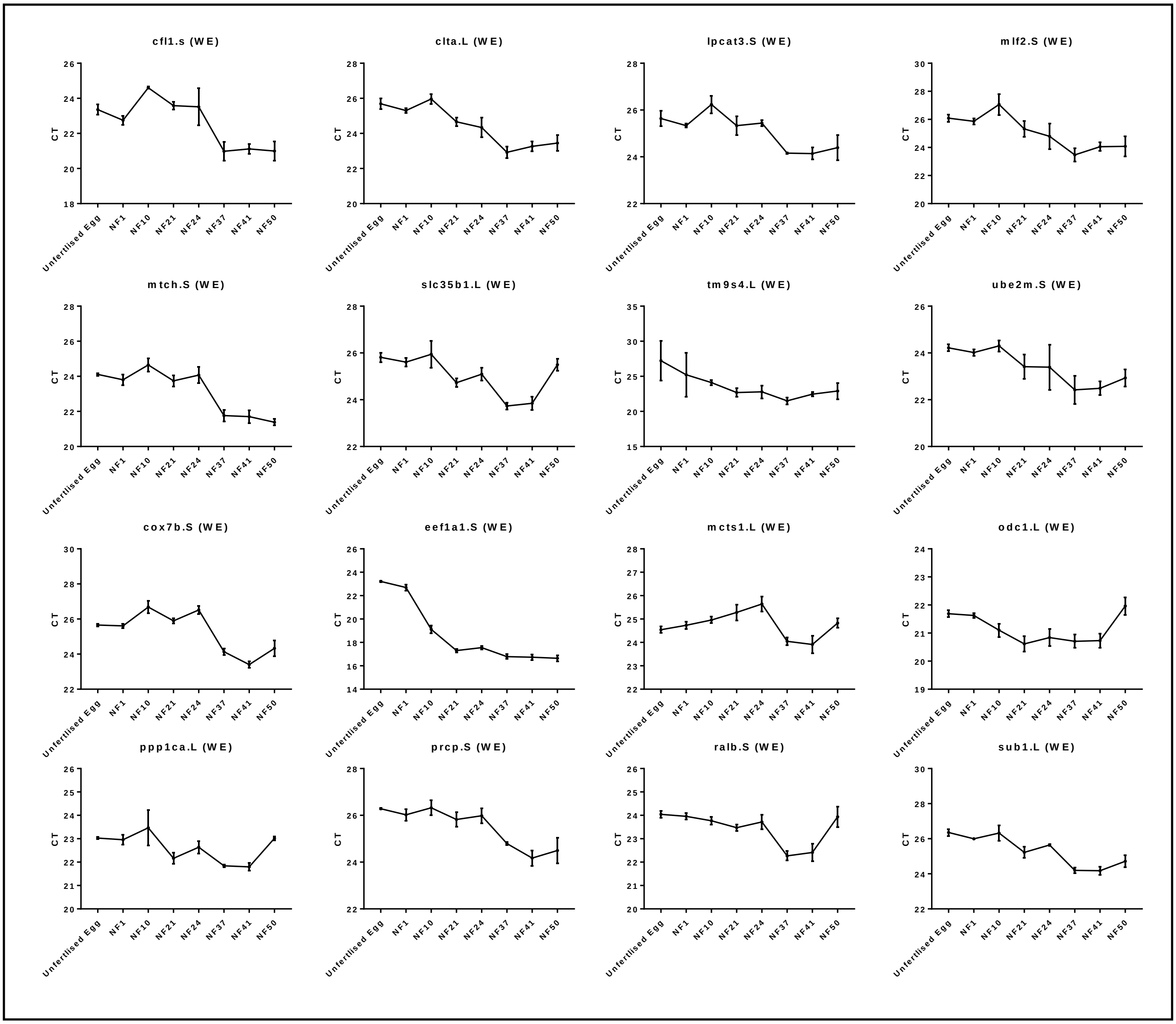

Supplementary figure 2c
Page 3/3

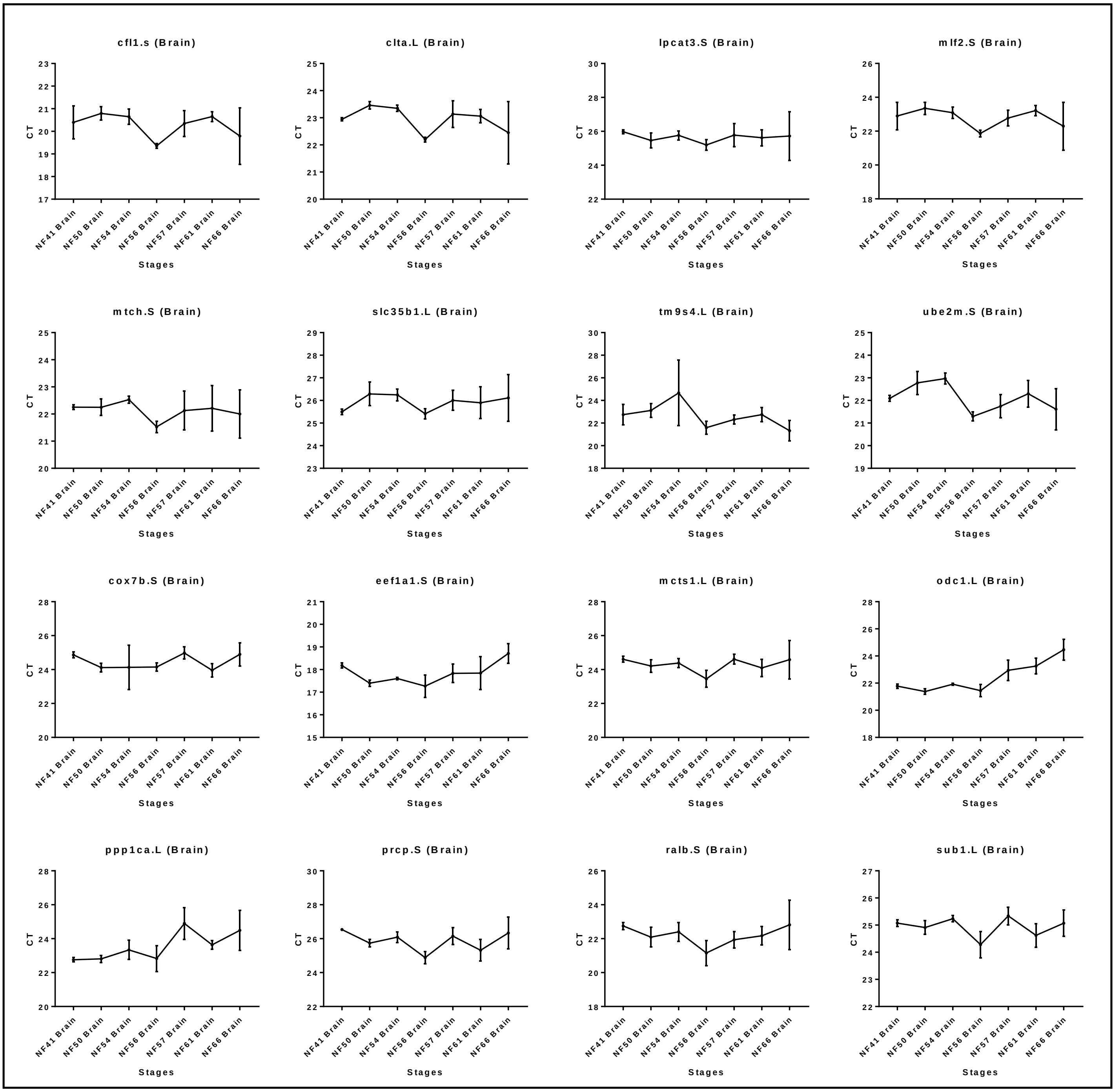

Supplementary figure 3

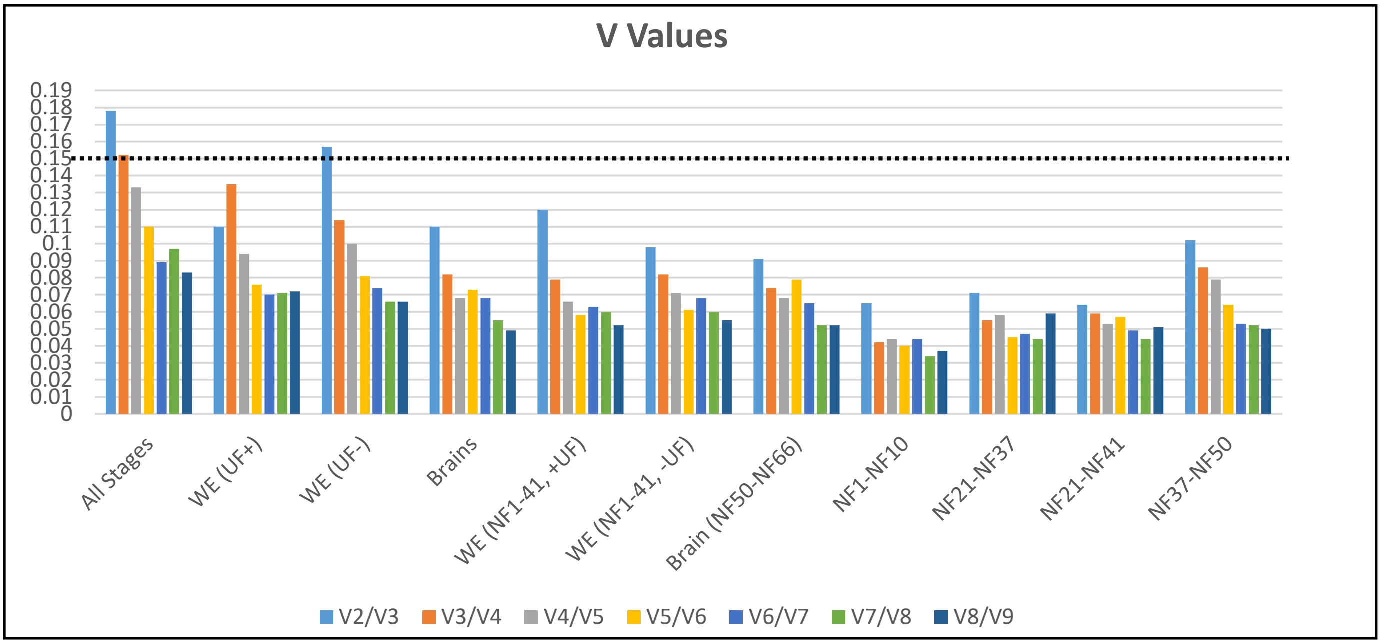

Supplementary figure 3: Pairwise Variation (V) analysis using geNorm of 16 reference genes in whole embryos during early developmental stages and in brain during metamorphic stages

Supplementary figure 4

Page 1/2

		geNorm		Detla CT		NormFinder		BestKeeper		
Sample group	Rank	Gene	Stability value	Genes	Average of STDEV	Gene	Stability Value	Gene	CV (%Ct)	SD (±Ct)
All samples (Whole embryos + brains)	1	clta.L	0.53	sub1.L	0.91	sub1.L	0.121	cfl1.S	6.32	1.36
	2	ube2m.S	0.543	mtch2.L	1.02	mtch2.L	0.502	clta.L	4.09	0.97
	3	mtch2.L	0.558	ube2m.S	1.05	ube2m.S	0.562	lpcat3.S	2.27	0.57
	4	sub1.L	0.623	mcts1.L	1.05	mcts1.L	0.598	mlf2.S	5.29	1.27
	5	cox7b.S	0.678	clta.L	1.05	clta.L	0.602	mtch2.L	4.28	0.97
	6	ralb.S	0.714	prcp.S	1.07	cox7b.S	0.627	slc35b1.L	2.46	0.63
	7	mcts1.L	0.728	cox7b.S	1.08	ralb.S	0.629	tm9s4.L	5.6	1.3
	8	prcp.S	0.769	lpcat3.S	1.08	prcp.S	0.636	ube2m.S	3.52	0.8
	9	lpcat3.S	0.793	ralb.S	1.09	lpcat3.S	0.675	cox7b.S	3.47	0.86
	10	slc35b1.L	0.836	slc35b1.L	1.2	slc35b1.L	0.862	eef1a1.S	7.63	1.4
	11	mlf2.S	0.899	ppp1ca.L	1.32	ppp1ca.L	1.054	mcts1.L	2.01	0.49
	12	cfl1.S	0.942	mlf2.S	1.32	mlf2.S	1.064	odc1.L	3.56	0.77
	13	ppp1ca.L	0.989	cfl1.S	1.39	cfl1.S	1.161	ppp1ca.L	2.96	0.68
	14	odc1.L	1.058	odc1.L	1.57	odc1.L	1.387	prcp.S	2.57	0.66
	15	eef1a1.S	1.155	eef1a1.S	1.77	eef1a1.S	1.547	ralb.S	3.71	0.85
	16	tm9s4.L	1.235	tm9s4.L	1.8	tm9s4.L	1.581	sub1.L	2.34	0.59
Whole Embryos (including unfertilised egg)	1	prcp.S	0.361	sub1.L	0.76	sub1.L	0.175	cfl1.S	5.3	1.2
	2	sub1.L	0.362	lpcat3.S	0.81	clta.L	0.335	clta.L	4.05	0.99
	3	lpcat3.S	0.366	prcp.S	0.83	ube2m.S	0.341	lpcat3.S	2.6	0.65
	4	slc35b1.L	0.468	ube2m.S	0.84	lpcat3.S	0.369	mlf2.S	4.24	1.06
	5	ppp1ca.L	0.497	clta.L	0.85	prcp.S	0.369	mtch2.L	4.98	1.15
	6	ube2m.S	0.517	slc35b1.L	0.86	slc35b1.L	0.428	slc35b1.L	2.86	0.72
	7	ralb.S	0.538	ppp1ca.L	0.91	mlf2.S	0.568	tm9s4.L	6.32	1.49
	8	clta.L	0.568	ralb.S	0.93	ppp1ca.L	0.569	ube2m.S	3.09	0.72
	9	mlf2.S	0.606	mtch2.L	0.96	ralb.S	0.593	cox7b.S	3.92	0.99
	10	cox7b.S	0.637	mlf2.S	0.96	mtch2.L	0.605	eef1a1.S	11.66	2.19
	11	mtch2.L	0.657	cox7b.S	0.97	cox7b.S	0.674	mcts1.L	1.86	0.46
	12	mcts1.L	0.683	mcts1.L	1.05	mcts1.L	0.826	odc1.L	2.2	0.47
	13	cfl1.S	0.712	cfl1.S	1.1	cfl1.S	0.851	ppp1ca.L	2.34	0.53
	14	odc1.L	0.748	odc1.L	1.12	odc1.L	0.876	prcp.S	2.94	0.75
	15	tm9s4.L	0.905	tm9s4.L	1.92	tm9s4.L	1.759	ralb.S	2.42	0.57
	16	eef1a1.S	1.066	eef1a1.S	2.19	eef1a1.S	2.081	sub1.L	3.04	0.77
Whole Embryos (minus unfertilised egg)	1	slc35b1.L	0.39	sub1.L	0.7	sub1.L	0.057	cfl1.S	5.64	1.27
	2	ppp1ca.L	0.404	lpcat3.S	0.76	lpcat3.S	0.338	clta.L	3.95	0.96
	3	sub1.L	0.435	clta.L	0.79	clta.L	0.352	lpcat3.S	2.68	0.67
	4	lpcat3.S	0.469	prcp.S	0.79	ube2m.S	0.373	mlf2.S	4.22	1.05
	5	prcp.S	0.502	ube2m.S	0.8	prcp.S	0.397	mtch2.L	5.22	1.2
	6	ube2m.S	0.53	slc35b1.L	0.81	slc35b1.L	0.446	slc35b1.L	2.87	0.71
	7	ralb.S	0.555	ppp1ca.L	0.85	ppp1ca.L	0.544	tm9s4.L	4.54	1.05
	8	mcts1.L	0.577	ralb.S	0.9	mlf2.S	0.61	ube2m.S	2.97	0.69
	9	clta.L	0.603	mlf2.S	0.93	ralb.S	0.613	cox7b.S	4.31	1.09
	10	cox7b.S	0.637	cox7b.S	0.93	cox7b.S	0.659	eef1a1.S	8.78	1.59
	11	mtch2.L	0.669	mtch2.L	0.93	mtch2.L	0.661	mcts1.L	1.99	0.49
	12	mlf2.S	0.693	mcts1.L	0.94	mcts1.L	0.686	odc1.L	2.05	0.43
	13	cfl1.S	0.728	cfl1.S	1.09	cfl1.S	0.898	ppp1ca.L	2.44	0.55
	14	odc1.L	0.769	odc1.L	1.11	odc1.L	0.914	prcp.S	2.99	0.76
	15	tm9s4.L	0.854	tm9s4.L	1.41	tm9s4.L	1.231	ralb.S	2.5	0.58
	16	eef1a1.S	0.97	eef1a1.S	1.79	eef1a1.S	1.674	sub1.L	2.89	0.73
Brains (NF41-NF66)	1	lpcat3.S	0.319	mcts1.L	0.59	mcts1.L	0.211	cfl1.S	2.98	0.61
	2	mcts1.L	0.331	mtch2.L	0.61	mtch2.L	0.237	clta.L	2.08	0.48
	3	mtch2.L	0.341	lpcat3.S	0.62	lpcat3.S	0.248	lpcat3.S	1.84	0.47
	4	sub1.L	0.359	sub1.L	0.62	sub1.L	0.257	mlf2.S	2.76	0.63
	5	slc35b1.L	0.378	slc35b1.L	0.64	slc35b1.L	0.306	mtch2.L	2.07	0.46
	6	clta.L	0.418	clta.L	0.68	clta.L	0.411	slc35b1.L	1.74	0.45
	7	cfl1.S	0.453	prcp.S	0.7	prcp.S	0.446	tm9s4.L	4.12	0.93
	8	mlf2.S	0.467	cfl1.S	0.72	ralb.S	0.467	ube2m.S	2.78	0.61
	9	ube2m.S	0.482	ralb.S	0.73	cfl1.S	0.471	cox7b.S	2.02	0.49
	10	prcp.S	0.505	mlf2.S	0.74	mlf2.S	0.503	eef1a1.S	2.62	0.47
	11	ralb.S	0.523	eef1a1.S	0.75	eef1a1.S	0.507	mcts1.L	1.86	0.45
	12	eef1a1.S	0.547	ube2m.S	0.76	ube2m.S	0.538	odc1.L	4.28	0.96
	13	cox7b.S	0.575	cox7b.S	0.81	cox7b.S	0.592	ppp1ca.L	3.12	0.73
	14	ppp1ca.L	0.627	ppp1ca.L	0.98	ppp1ca.L	0.823	prcp.S	2.12	0.55
	15	odc1.L	0.691	odc1.L	1.17	odc1.L	1.071	ralb.S	2.62	0.58
	16	tm9s4.L	0.786	tm9s4.L	1.45	tm9s4.L	1.377	sub1.L	1.38	0.34

Supplementary figure 4: Comparison of 4 different statistical algorithms used to calculate reference gene stability. Four different statistical algorithms, geNorm, delta-CT, NormFinder and BestKeeper, were used to compare the gene expression and stability of the 16 candidate reference genes.

Supplementary figure 4

Page 1/2

Supplementary

		geNorm		Delta CT		NormFinder		BestKeeper		
Sample group	Rank	Gene	Stability value	Genes	Average of STDEV	Gene	Stability Value	Gene	CV (%Ct)	SD (±Ct)
WE (NF1 - 41 including Unfertilised egg)	1	slc35b1.L	0.252	slc35b1.L	0.7	sub1.L	0.033	cfl1.S	4.72	1.08
	2	sub1.L	0.296	sub1.L	0.7	slc35b1.L	0.085	clta.L	3.94	0.97
	3	prcp.S	0.303	lpcat3.S	0.74	clta.L	0.313	lpcat3.S	2.44	0.61
	4	lpcat3.S	0.333	prcp.S	0.76	ube2m.S	0.337	mlf2.S	4.16	1.05
	5	ralb.S	0.356	ralb.S	0.78	prcp.S	0.349	mtch.2	4.09	0.96
	6	ppp1ca.L	0.375	ube2m.S	0.79	lpcat3.S	0.355	slc35b1.L	3.03	0.76
	7	ube2m.S	0.412	mtch.2	0.79	ralb.S	0.362	tm9s4.L	6.75	1.6
	8	mtch.2	0.447	ppp1ca.L	0.8	mtch.2	0.407	ube2m.S	3.22	0.75
	9	clta.L	0.469	clta.L	0.8	ppp1ca.L	0.426	cox7b.S	3.7	0.94
	10	mlf2.S	0.506	mlf2.S	0.92	mlf2.S	0.561	eef1a1.S	11.91	2.27
	11	cox7b.S	0.541	cox7b.S	0.95	odc1.L	0.704	mcts1.L	2.02	0.5
	12	cfl1.S	0.577	odc1.L	0.99	cox7b.S	0.709	odc1.L	1.83	0.39
	13	mcts1.L	0.609	mcts1.L	1.02	cfl1.S	0.793	ppp1ca.L	2.44	0.55
	14	odc1.L	0.639	cfl1.S	1.02	mcts1.L	0.827	prcp.S	2.61	0.67
	15	tm9s4.L	0.822	tm9s4.L	1.99	tm9s4.L	1.86	ralb.S	2.54	0.59
	16	eef1a1.S	0.997	eef1a1.S	2.22	eef1a1.S	2.129	sub1.L	3.04	0.77
Sample group	Rank	Gene	Stability value	Genes	Average of STDEV	Gene	Stability Value	Gene	CV (%Ct)	SD (±Ct)
WE (NF1 - 41 minus Unfertilised egg)	1	slc35b1.L	0.231	sub1.L	0.64	slc35b1.L	0.089	cfl1.S	5.19	1.18
	2	sub1.L	0.242	slc35b1.L	0.64	sub1.L	0.096	clta.L	3.89	0.95
	3	lpcat3.S	0.263	lpcat3.S	0.67	lpcat3.S	0.303	lpcat3.S	2.62	0.66
	4	ppp1ca.L	0.312	prcp.S	0.71	clta.L	0.333	mlf2.S	4.32	1.08
	5	prcp.S	0.348	ralb.S	0.73	prcp.S	0.366	mtch2.L	4.46	1.04
	6	ralb.S	0.374	clta.L	0.74	ube2m.S	0.375	slc35b1.L	2.97	0.74
	7	ube2m.S	0.419	ppp1ca.L	0.74	ralb.S	0.385	tm9sf4.L	4.76	1.1
	8	clta.L	0.453	ube2m.S	0.75	ppp1ca.L	0.402	ube2m.S	3.21	0.75
	9	mtch2.L	0.48	mtch2.L	0.76	mtch2.L	0.44	cox7b.S	4.22	1.07
	10	mcts1.L	0.517	mlf2.S	0.89	mlf2.S	0.606	eef1a1.S	9.22	1.69
	11	cox7b.S	0.549	mcts1.L	0.9	cox7b.S	0.684	mcts1.L	2.21	0.55
	12	mlf2.S	0.584	cox7b.S	0.9	mcts1.L	0.696	odc1.L	1.49	0.31
	13	cfl1.S	0.62	odc1.L	0.98	odc1.L	0.755	ppp1ca.L	2.47	0.56
	14	odc1.L	0.656	cfl1.S	1	cfl1.S	0.823	prcp.S	2.73	0.7
	15	tm9sf4.L	0.76	tm9sf4.L	1.44	tm9sf4.L	1.287	ralb.S	2.65	0.62
	16	eef1a1.S	0.894	eef1a1.S	1.83	eef1a1.S	1.745	sub1.L	3.02	0.76
Sample group	Rank	Gene	Stability value	Genes	Average of STDEV	Gene	Stability Value	Gene	CV (%Ct)	SD (±Ct)
Brain (NF50-66)	1	slc35b1.L	0.31	mcts1.L	0.59	mcts1.L	0.156	cfl1.S	3.08	0.62
	2	mcts1.L	0.311	slc35b1.L	0.6	slc35b1.L	0.225	clta.L	2.4	0.55
	3	lpcat3.S	0.312	mtch2.L	0.62	lpcat3.S	0.246	lpcat3.S	1.91	0.49
	4	mtch2.L	0.328	lpcat3.S	0.63	mtch2.L	0.247	mlf2.S	2.82	0.64
	5	sub1.L	0.354	sub1.L	0.63	sub1.L	0.279	mtch2.L	2.33	0.51
	6	clta.L	0.41	prcp.S	0.68	prcp.S	0.389	slc35b1.L	1.74	0.45
	7	cfl1.S	0.439	clta.L	0.7	clta.L	0.443	tm9sf4.L	4.31	0.97
	8	mlf2.S	0.449	cfl1.S	0.72	ralb.S	0.451	ube2m.S	3.17	0.7
	9	prcp.S	0.47	ralb.S	0.73	cfl1.S	0.476	cox7b.S	2.04	0.5
	10	ube2m.S	0.492	mlf2.S	0.74	mlf2.S	0.5	eef1a1.S	2.64	0.47
	11	ralb.S	0.513	eef1a1.S	0.77	eef1a1.S	0.532	mcts1.L	1.93	0.47
	12	eef1a1.S	0.543	ube2m.S	0.8	ube2m.S	0.583	odc1.L	4.52	1.02
	13	cox7b.S	0.577	cox7b.S	0.84	cox7b.S	0.622	ppp1ca.L	3.01	0.71
	14	ppp1ca.L	0.623	ppp1ca.L	0.96	ppp1ca.L	0.791	prcp.S	2.05	0.53
	15	odc1.L	0.69	odc1.L	1.2	odc1.L	1.102	ralb.S	2.54	0.56
	16	tm9sf4.L	0.797	tm9sf4.L	1.54	tm9sf4.L	1.474	sub1.L	1.51	0.38
Sample group	Rank	Gene	Stability value	Genes	Average of STDEV	Gene	Stability Value	Gene	CV (%Ct)	SD (±Ct)
NF1-NF10	1	ube2m.S	0.131	prcp.S	0.6	ube2m.S	0.044	cfl1.S	3.92	0.93
	2	mcts1.L	0.156	sub1.L	0.6	sub1.L	0.07	clta.L	1.29	0.33
	3	prcp.S	0.17	ube2m.S	0.61	slc35b1.L	0.07	lpcat3.S	1.75	0.45
	4	sub1.L	0.182	clta.L	0.62	prcp.S	0.073	mlf2.S	2.32	0.61
	5	slc35b1.L	0.204	slc35b1.L	0.62	mcts1.L	0.213	mtch2.L	1.75	0.42
	6	clta.L	0.227	mtch2.L	0.65	clta.L	0.239	slc35b1.L	1.11	0.28
	7	mtch2.L	0.258	mcts1.L	0.65	mtch2.L	0.319	tm9sf4.L	5.61	1.38
	8	lpcat3.S	0.273	lpcat3.S	0.65	ppp1ca.L	0.322	ube2m.S	0.64	0.15
	9	ppp1ca.L	0.295	ppp1ca.L	0.7	ralb.S	0.356	cox7b.S	2.06	0.54
	10	cox7b.S	0.316	cox7b.S	0.73	lpcat3.S	0.384	eef1a1.S	8.56	1.79
	11	mlf2.S	0.345	ralb.S	0.76	odc1.L	0.414	mcts1.L	0.53	0.13
	12	ralb.S	0.38	mlf2.S	0.81	cox7b.S	0.508	odc1.L	1.24	0.26
	13	odc1.L	0.419	odc1.L	0.84	mlf2.S	0.618	ppp1ca.L	1.89	0.44
	14	cfl1.S	0.477	cfl1.S	1.08	cfl1.S	0.98	prcp.S	0.77	0.2
	15	tm9sf4.L	0.7	tm9sf4.L	2.16	tm9sf4.L	2.048	ralb.S	0.48	0.12
	16	eef1a1.S	0.897	eef1a1.S	2.28	eef1a1.S	2.199	sub1.L	0.89	0.23
Sample group	Rank	Gene	Stability value	Genes	Average of STDEV	Gene	Stability Value	Gene	CV (%Ct)	SD (±Ct)
NF21 - NF37		sub1.L	0.209	slc35b1.L	0.46	slc35b1.L	0.135	cfl1.S	5.04	1.14
		slc35b1.L	0.219	sub1.L	0.48	lpcat3.S	0.2	clta.L	3.13	0.75
		lpcat3.S	0.224	lpcat3.S	0.48	sub1.L	0.22	lpcat3.S	2.22	0.55
		mcts1.L	0.239	mcts1.L	0.5	mcts1.L	0.227	mlf2.S	3.5	0.86
		prcp.S	0.272	ralb.S	0.53	ralb.S	0.324	mtch2.L	4.11	0.95
		ralb.S	0.288	prcp.S	0.55	clta.L	0.356	slc35b1.L	2.13	0.52
		eef1a1.S	0.313	eef1a1.S	0.57	prcp.S	0.368	tm9sf4.L	3.21	0.72
		ppp1ca.L	0.335	clta.L	0.57	eef1a1.S	0.417	ube2m.S	2.52	0.58
		cox7b.S	0.385	ppp1ca.L	0.59	tm9sf4.L	0.436	cox7b.S	3.6	0.92
		clta.L	0.425	tm9sf4.L	0.62	ppp1ca.L	0.44	eef1a1.S	1.76	0.3
		mtch2.L	0.459	mtch2.L	0.64	mtch2.L	0.484	mcts1.L	2.55	0.64
		tm9sf4.L	0.488	cox7b.S	0.68	ube2m.S	0.528	odc1.L	1.01	0.21
		ube2m.S	0.515	ube2m.S	0.68	cox7b.S	0.538	ppp1ca.L	1.45	0.32
		mlf2.S	0.537	mlf2.S	0.69	mlf2.S	0.551	prcp.S	1.98	0.5
		odc1.L	0.568	odc1.L	0.82	odc1.L	0.75	ralb.S	2.55	0.59
		cfl1.S	0.61	cfl1.S	0.9	cfl1.S	0.831	sub1.L	2.23	0.56
Sample group	Rank	Gene	Stability value	Genes	Average of STDEV	Gene	Stability Value	Gene	CV (%Ct)	SD (±Ct)
NF21-NF41		sub1.L	0.192	slc35b1.L	0.47	slc35b1.L	0.126	cfl1.S	5.6	1.25
		lpcat3.S	0.201	sub1.L	0.48	lpcat3.S	0.159	clta.L	2.99	0.71
		slc35b1.L	0.205	lpcat3.S	0.48	sub1.L	0.175	lpcat3.S	2.5	0.62
		mcts1.L	0.233	mcts1.L	0.51	mcts1.L	0.224	mlf2.S	3.04	0.74
		ralb.S	0.26	ralb.S	0.52	ralb.S	0.274	mtch2.L	4.77	1.09
		eef1a1.S	0.299	clta.L	0.55	clta.L	0.302	slc35b1.L	2.3	0.56
		ppp1ca.L	0.323	eef1a1.S	0.56	eef1a1.S	0.389	tm9sf4.L	2.7	0.6
		prcp.S	0.345	ppp1ca.L	0.59	prcp.S	0.402	ube2m.S	2.25	0.52
		clta.L	0.381	prcp.S	0.59	ppp1ca.L	0.422	cox7b.S	4.88	1.22
		ube2m.S	0.424	ube2m.S	0.65	ube2m.S	0.468	eef1a1.S	1.95	0.33
		mlf2.S	0.458	mlf2.S	0.66	mlf2.S	0.495	mcts1.L	2.99	0.74
		tm9sf4.L	0.487	mtch2.L	0.68	mtch2.L	0.521	odc1.L	0.99	0.2
		mtch2.L	0.515	tm9sf4.L	0.72	tm9sf4.L	0.589	ppp1ca.L	1.45	0.32
		odc1.L	0.548	odc1.L	0.83	odc1.L	0.754	prcp.S	2.82	0.71
		cox7b.S	0.591	cox7b.S	0.86	cox7b.S	0.771	ralb.S	2.74	0.63
		cfl1.S	0.627	cfl1.S	0.88	cfl1.S	0.793	sub1.L	2.5	0.62
Sample group	Rank	Gene	Stability value	Genes	Average of STDEV	Gene	Stability Value	Gene	CV (%Ct)	SD (±Ct)
NF37-NF50		lpcat3.S	0.278	sub1.L	0.46	sub1.L	0.148	cfl1.S	1.68	0.35
		eef1a1.S	0.297	clta.L	0.5	clta.L	0.245	clta.L	1.27	0.29
		sub1.L	0.309	mcts1.L	0.52	mcts1.L	0.282	lpcat3.S	0.91	0.22
		prcp.S	0.349	ppp1ca.L	0.52	ppp1ca.L	0.324	mlf2.S	1.73	0.41
		mtch2.L	0.387	lpcat3.S	0.55	lpcat3.S	0.361	mtch2.L	1.15	0.25
		cfl1.S	0.409	odc1.L	0.55	odc1.L	0.366	slc35b1.L	3.11	0.76
		clta.L	0.42	ube2m.S	0.58	ube2m.S	0.379	tm9sf4.L	3.12	0.7
		cox7b.S	0.438	eef1a1.S	0.58	cox7b.S	0.4	ube2m.S	1.57	0.35
		mcts1.L	0.457	cox7b.S	0.58	eef1a1.S	0.424	cox7b.S	1.64	0.39
		ube2m.S	0.473	cfl1.S	0.59	cfl1.S	0.432	eef1a1.S	1.02	0.17
		mlf2.S	0.486	mlf2.S	0.6	mlf2.S	0.435	mcts1.L	1.55	0.38
		ppp1ca.L	0.5	prcp.S	0.62	prcp.S	0.481	odc1.L	2.61	0.55
		odc1.L	0.511	mtch2.L	0.64	mtch2.L	0.522	ppp1ca.L	2.4	0.53
		slc35b1.L	0.541	slc35b1.L	0.71	slc35b1.L	0.606	prcp.S	1.36	0.33
		ralb.S	0.566	ralb.S	0.74	ralb.S	0.644	ralb.S	3.11	0.71
		tm9sf4.L	0.595	tm9sf4.L	0.8	tm9sf4.L	0.699	sub1.L	1	0.24

A

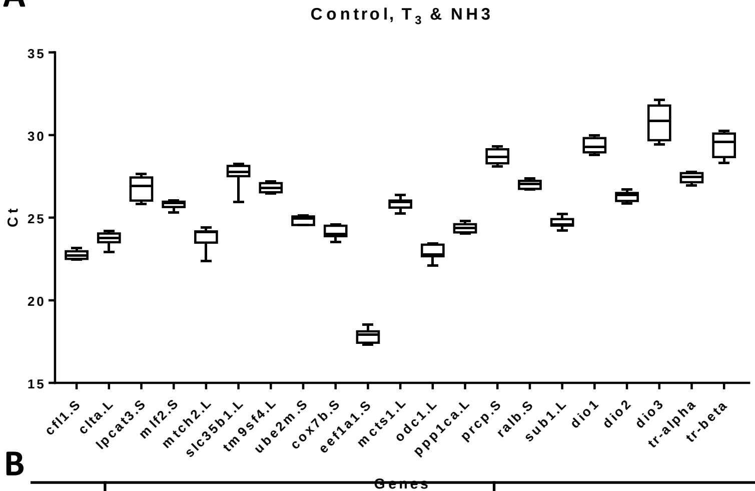

B

	Ctrl + T3			Ctrl + T3 + NH3			Ctrl + T3 + NH3 + TCS		
**Rank**	**Gene**	**Mean**	**Std Deviation**	**Gene**	**Mean**	**Std Deviation**	**Gene**	**Mean**	**Std Deviation**
**1**	mlf2.S	25.9	0.1287	mlf2.S	25.8	0.2377	ppp1ca.L	24.4	0.243
**2**	mtch2.L	24.15	0.1717	ralb.S	27.02	0.2476	ralb.S	27.03	0.2525
**3**	eef1a1.S	17.94	0.2055	cfl1.S	22.75	0.2548	ube2m.S	24.86	0.2614
**4**	ube2m.S	24.91	0.2093	ube2m.S	24.85	0.256	mlf2.S	25.79	0.2719
**5**	clta.L	23.91	0.2316	tm9sf4.L	26.82	0.275	dio2	26.31	0.2926
**6**	cfl1.S	22.82	0.2352	dio2	26.3	0.287	sub1.L	24.7	0.2974
**7**	mcts1.L	26.01	0.2364	ppp1ca.L	24.38	0.2883	cfl1.S	22.74	0.3107
**8**	ralb.S	27.09	0.243	sub1.L	24.68	0.3118	tr-alpha	27.41	0.3364
**9**	tr-alpha	27.58	0.244	tr-alpha	27.42	0.3124	cox7b.S	24.14	0.3676
**10**	tm9sf4.L	26.93	0.2899	mcts1.L	25.87	0.3401	mcts1.L	25.79	0.3691
**11**	prcp.S	28.96	0.3111	cox7b.S	24.12	0.3737	clta.L	23.71	0.3972
**12**	ppp1ca.L	24.45	0.3177	clta.L	23.71	0.3992	tm9sf4.L	26.84	0.4194
**13**	slc35b1.L	27.9	0.3477	eef1a1.S	17.86	0.4075	eef1a1.S	17.79	0.4339
**14**	dio2	26.26	0.3611	prcp.S	28.7	0.4439	prcp.S	28.67	0.4374
**15**	sub1.L	24.68	0.386	odc1.L	22.89	0.4563	odc1.L	22.94	0.4613
**16**	cox7b.S	24.09	0.4203	dio1	29.35	0.4692	dio1	29.16	0.5635
**17**	dio1	29.48	0.4934	mtch2.L	23.83	0.6602	mtch2.L	23.86	0.5951
**18**	odc1.L	22.87	0.5279	lpcat3.S	26.79	0.7035	slc35b1.L	27.67	0.6362
**19**	lpcat3.S	26.82	0.7009	tr-beta	29.4	0.7151	lpcat3.S	26.87	0.6453
**20**	tr-beta	29.26	0.9108	slc35b1.L	27.62	0.7326	tr-beta	29.65	0.7532
**21**	dio3	30.44	1.163	dio3	30.75	1.048	dio3	30.9	0.9173

Supplementary figure 5: Ct values of 16 reference genes in NF48 brain exposed to thyroid (T_3). Variation of 16 reference genes mRNA expression (Ct) assessed using RT-qPCR **(A)** Control vs T3 vs NH3 (T3 antagonist). **(B)** Variation of 16 candidate genes RNA expression during different series of experimental conditions Control vs T3, Control vs T3 vs NH3 (T3 antagonist) and, Control vs T3 vs NH3 (T3 antagonist) vs Triclosan. **(C)** Analysis using four different statistical algorithms, geNorm, delta-CT, NormFinder and BestKeeper on series of different experimental conditions.

Supplementary figure 5c- Page 2/3

C

		geNorm		Delta CT		NormFinder		BestKeeper		
**Sample group**	**Rank**	**Gene**	**Stability value**	**Genes**	**Average of STDEV**	**Gene**	**Stability Value**	**Gene**	**CV (%Ct)**	**SD (±Ct)**
Control + T3 + NH3	1	ralb.S	0.12	ube2m.S	0.35	cfl1.S	0.022	cfl1.S	0.95	0.22
	2	ube2m.S	0.122	cfl1.S	0.36	ube2m.S	0.057	clta.L	1.12	0.27
	3	mlf2.S	0.126	ralb.S	0.36	ralb.S	0.125	lpcat3.S	2.22	0.6
	4	cfl1.S	0.139	mlf2.S	0.38	sub1.L	0.158	mlf2.S	0.72	0.19
	5	tm9sf4.L	0.161	tm9sf4.L	0.39	tm9sf4.L	0.171	mtch2.L	2.02	0.48
	6	ppp1ca.L	0.169	sub1.L	0.39	mlf2.S	0.183	slc35b1.L	1.69	0.47
	7	sub1.L	0.186	ppp1ca.L	0.4	ppp1ca.L	0.188	tm9sf4.L	0.84	0.23
	8	cox7b.S	0.204	eef1a1.S	0.41	cox7b.S	0.203	ube2m.S	0.9	0.22
	9	dio2	0.219	cox7b.S	0.41	eef1a1.S	0.208	cox7b.S	1.27	0.31
	10	odc1.L	0.232	mcts1.L	0.43	odc1.L	0.248	eef1a1.S	1.79	0.32
	11	eef1a1.S	0.246	clta.L	0.43	clta.L	0.249	mcts1.L	0.98	0.25
	12	mcts1.L	0.257	odc1.L	0.44	mcts1.L	0.27	odc1.L	1.58	0.36
	13	clta.L	0.272	dio2	0.46	dio2	0.306	ppp1ca.L	1.05	0.26
	14	tr-alpha	0.294	prcp.S	0.52	prcp.S	0.405	prcp.S	1.27	0.37
	15	prcp.S	0.31	tr-alpha	0.53	tr-alpha	0.434	ralb.S	0.74	0.2
	16	dio1	0.336	mtch2.L	0.6	mtch2.L	0.489	sub1.L	0.98	0.24
	17	mtch2.L	0.362	dio1	0.62	lpcat3.S	0.533	dio1	1.41	0.41
	18	slc35b1.L	0.39	slc35b1.L	0.65	slc35b1.L	0.541	dio2	0.84	0.22
	19	lpcat3.S	0.415	lpcat3.S	0.65	dio1	0.548	dio3	2.9	0.89
	20	tr-beta	0.45	tr-beta	0.76	tr-beta	0.677	tr-alpha	0.99	0.27
	21	dio3	0.502	dio3	0.99	dio3	0.951	tr-beta	2	0.59
**Sample group**	**Rank**	**Gene**	**Stability value**	**Genes**	**Average of STDEV**	**Gene**	**Stability Value**	**Gene**	**CV (%Ct)**	**SD (±Ct)**
Control + T3 + NH + TCS	1	ube2m.S	0.125	ube2m.S	0.34	cfl1.S	0.057	cfl1.S	1.15	0.26
	2	ralb.S	0.127	cfl1.S	0.34	ube2m.S	0.065	clta.L	1.23	0.29
	3	mlf2.S	0.128	ralb.S	0.35	ralb.S	0.118	lpcat3.S	2.08	0.56
	4	cfl1.S	0.14	mlf2.S	0.35	sub1.L	0.142	mlf2.S	0.86	0.22
	5	sub1.L	0.167	sub1.L	0.37	mlf2.S	0.155	mtch2.L	1.87	0.45
	6	cox7b.S	0.184	cox7b.S	0.38	cox7b.S	0.169	slc35b1.L	1.47	0.41
	7	dio2	0.199	clta.L	0.39	clta.L	0.209	tm9sf4.L	1.21	0.32
	8	ppp1ca.L	0.21	eef1a1.S	0.41	odc1.L	0.226	ube2m.S	0.94	0.23
	9	odc1.L	0.223	ppp1ca.L	0.41	ppp1ca.L	0.229	cox7b.S	1.32	0.32
	10	eef1a1.S	0.241	odc1.L	0.41	eef1a1.S	0.241	eef1a1.S	1.92	0.34
	11	mcts1.L	0.254	mcts1.L	0.42	dio2	0.254	mcts1.L	1.12	0.29
	12	clta.L	0.264	dio2	0.42	tm9sf4.L	0.269	odc1.L	1.74	0.4
	13	tm9sf4.L	0.273	tm9sf4.L	0.43	mcts1.L	0.278	ppp1ca.L	0.81	0.2
	14	prcp.S	0.289	prcp.S	0.47	prcp.S	0.352	prcp.S	1.26	0.36
	15	tr-alpha	0.301	tr-alpha	0.49	tr-alpha	0.379	ralb.S	0.76	0.21
	16	mtch2.L	0.323	mtch2.L	0.52	mtch2.L	0.41	sub1.L	0.97	0.24
	17	slc35b1.L	0.347	slc35b1.L	0.57	slc35b1.L	0.462	dio1	1.49	0.43
	18	lpcat3.S	0.369	lpcat3.S	0.58	lpcat3.S	0.468	dio2	0.91	0.24
	19	dio1	0.391	dio1	0.64	dio1	0.584	dio3	2.46	0.76
	20	tr-beta	0.431	tr-beta	0.78	tr-beta	0.711	tr-alpha	1.07	0.29
	21	dio3	0.475	dio3	0.89	dio3	0.842	tr-beta	1.97	0.58
**Sample group**	**Rank**	**Gene**	**Stability value**	**Genes**	**Average of STDEV**	**Gene**	**Stability Value**	**Gene**	**CV (%Ct)**	**SD (±Ct)**
Control + T3	1	eef1a1.S	0.091	ube2m.S	0.33	cfl1.S	0.047	cfl1.S	0.77	0.18
	2	ube2m.S	0.096	cfl1.S	0.34	ube2m.S	0.079	clta.L	0.84	0.2
	3	mlf2.S	0.098	eef1a1.S	0.34	sub1.L	0.119	lpcat3.S	2.16	0.58
	4	ralb.S	0.12	mlf2.S	0.34	slc35b1.L	0.123	mlf2.S	0.37	0.09
	5	clta.L	0.138	ralb.S	0.35	eef1a1.S	0.136	mtch2.L	0.46	0.11
	6	cfl1.S	0.15	slc35b1.L	0.37	ralb.S	0.141	slc35b1.L	1.03	0.29
	7	mtch2.L	0.161	clta.L	0.37	mlf2.S	0.158	tm9sf4.L	0.87	0.23
	8	slc35b1.L	0.182	sub1.L	0.37	cox7b.S	0.166	ube2m.S	0.58	0.14
	9	sub1.L	0.199	mtch2.L	0.37	mtch2.L	0.174	cox7b.S	1.37	0.33
	10	tm9sf4.L	0.211	tm9sf4.L	0.38	tm9sf4.L	0.185	eef1a1.S	0.81	0.15
	11	cox7b.S	0.221	cox7b.S	0.38	clta.L	0.201	mcts1.L	0.59	0.15
	12	ppp1ca.L	0.23	ppp1ca.L	0.4	ppp1ca.L	0.224	odc1.L	1.72	0.39
	13	dio2	0.239	dio2	0.41	dio2	0.232	ppp1ca.L	1.08	0.26
	14	mcts1.L	0.248	mcts1.L	0.41	odc1.L	0.28	prcp.S	0.85	0.25
	15	prcp.S	0.261	odc1.L	0.45	mcts1.L	0.284	ralb.S	0.66	0.18
	16	odc1.L	0.273	prcp.S	0.46	prcp.S	0.351	sub1.L	1.18	0.29
	17	tr-alpha	0.29	tr-alpha	0.51	tr-alpha	0.436	dio1	1.39	0.41
	18	lpcat3.S	0.329	lpcat3.S	0.68	lpcat3.S	0.592	dio2	1.11	0.29
	19	dio1	0.364	dio1	0.75	dio1	0.719	dio3	3.18	0.97
	20	tr-beta	0.41	tr-beta	0.81	tr-beta	0.748	tr-alpha	0.61	0.17
	21	dio3	0.468	dio3	1.02	dio3	0.99	tr-beta	2.65	0.78
**Sample group**	**Rank**	**Gene**	**Stability value**	**Genes**	**Average of STDEV**	**Gene**	**Stability Value**	**Gene**	**CV (%Ct)**	**SD (±Ct)**
Control + NH3	1	ube2m.S	0.054	cfl1.S	0.26	cfl1.S	0.042	cfl1.S	1.14	0.26
	2	cox7b.S	0.055	ube2m.S	0.27	odc1.L	0.048	clta.L	1.46	0.35
	3	cfl1.S	0.067	cox7b.S	0.27	ube2m.S	0.094	lpcat3.S	2.05	0.55
	4	ralb.S	0.085	odc1.L	0.27	cox7b.S	0.1	mlf2.S	0.88	0.23
	5	sub1.L	0.096	ralb.S	0.28	sub1.L	0.128	mtch2.L	2.79	0.66
	6	mlf2.S	0.104	sub1.L	0.28	ralb.S	0.134	slc35b1.L	2.37	0.65
	7	odc1.L	0.113	mlf2.S	0.3	clta.L	0.171	tm9sf4.L	0.85	0.23
	8	mcts1.L	0.122	mcts1.L	0.31	tm9sf4.L	0.178	ube2m.S	1.02	0.25
	9	ppp1ca.L	0.134	tm9sf4.L	0.31	mlf2.S	0.187	cox7b.S	1.09	0.27
	10	tm9sf4.L	0.145	ppp1ca.L	0.32	mcts1.L	0.194	eef1a1.S	2.27	0.41
	11	dio2	0.165	clta.L	0.34	ppp1ca.L	0.196	mcts1.L	1.12	0.29
	12	tr-alpha	0.183	prcp.S	0.37	eef1a1.S	0.256	odc1.L	1.42	0.33
	13	tr-beta	0.2	eef1a1.S	0.38	prcp.S	0.259	ppp1ca.L	1	0.25
	14	prcp.S	0.214	dio2	0.38	tr-beta	0.309	prcp.S	1.08	0.31
	15	clta.L	0.227	tr-beta	0.4	dio2	0.314	ralb.S	0.79	0.21
	16	eef1a1.S	0.243	tr-alpha	0.41	dio1	0.341	sub1.L	0.83	0.21
	17	dio1	0.257	dio1	0.43	tr-alpha	0.361	dio1	0.71	0.21
	18	dio3	0.283	dio3	0.49	dio3	0.396	dio2	0.47	0.13
	19	lpcat3.S	0.309	lpcat3.S	0.51	lpcat3.S	0.434	dio3	1.59	0.5
	20	mtch2.L	0.337	mtch2.L	0.57	mtch2.L	0.52	tr-alpha	0.69	0.19
	21	slc35b1.L	0.374	slc35b1.L	0.72	slc35b1.L	0.694	tr-beta	0.97	0.29

Supplementary figure 5d- Page 3/3

D

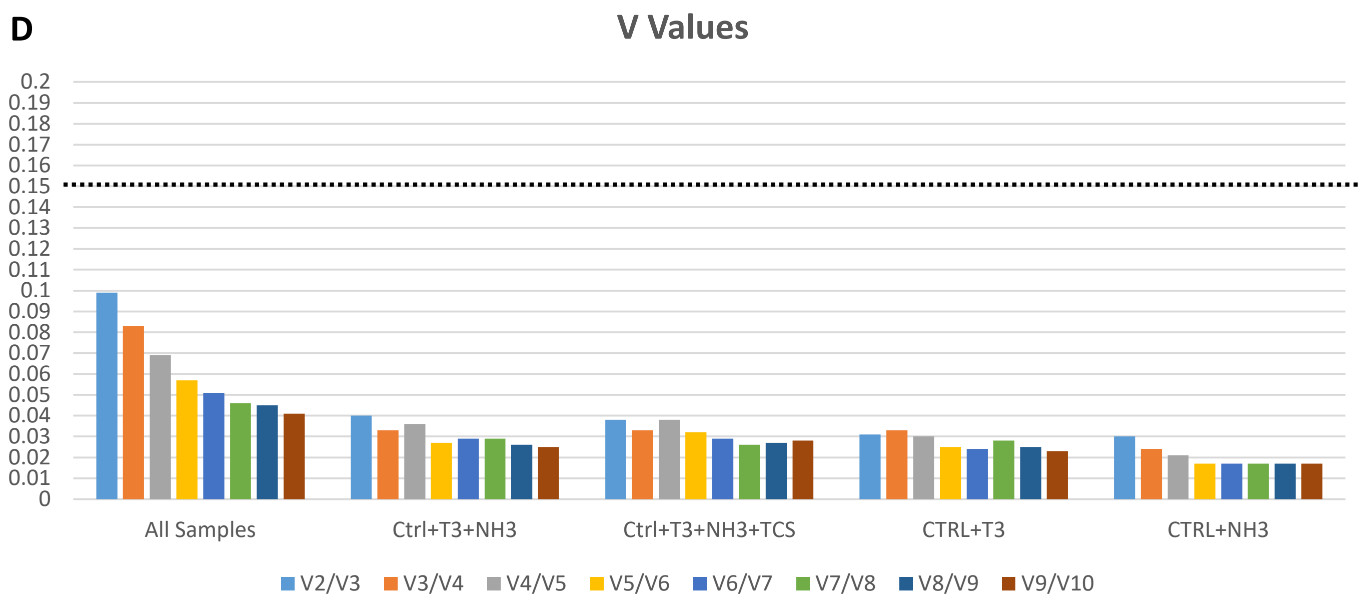

Supplementary table 1

Primers for gene expression (RT-qPCR)

Gene	Forward primer	Reverse primer	Exon	Length (bp)	Efficiency	Slope (R^2)
clta.L	GGGATGGGGCCTCAGATACT	GTCAGCGTGTGAAATGGCTG	E2-E3J	92	93.599	0.984
cfl1.S	TCGTTCTCTCGCTGAAGACTC	CCATCACACCAGAGGCCATTTT	E1-E2J	88	102.668	0.996
mlf2.S	CATGCGGCACATGTTCTCTG	GTCACGGCTCCTTGCTGTAT	E4-E5J	120	104.016	0.981
tm9sf4.L	TGAGGTTGTGTGCGGGA	GGTTATCCGCTATCAGGTGAA	E4-E5J	114	93.713	0.995
lpcat3.S	CAGTGATGATTGTACACCACTCC	ATGCCCTTCCCTCAAGTCAA	E13J-UTR	122	121.422	0.947
slc35b1.L	CGCATTTCCAAACAGGCTCC	CAAGAAGTCCCAGAGCTCGC	E7-E8	107	97.972	0.932
mtch2.L	TCGCACCCGATGATGTATGT	TGATGTGTTTGGCATAGGAGA	E2-E3J	133	100.694	0.994
ube2m.S	AGAGAGGACTGGAAGCCTGT	CGGGTCTTCAGGATTTGGCT	E5-E6	87	91.468	0.964
ppp1ca.L	ACGAGTCTCTCATGTGCTCC	CAGAGCTGGGAGGGGTCATT	E4-E5	140	92.59	0.997
sub1.L	AGCAGGAGAAATGAAGCCAGG	CCGACATCTGCTCCTTCAGT	E4-E5	80	97.972	0.973
mcts1.L	CACTGAGACTGCTTCACAAATATCC	TGATGTTAGCGCCACTCAGG	E4J-E5	106	116.719	0.956
ralb.S	CCACTGCCGAGTTCAGGGAA	CCAGATCGGATTTGTTGCCC	E4J-E5	90	107.707	0.985
cox7b.S	GGGTTATGTTATTACACAGACTGG C	ACAGGATTAACGCATAAAAAGGTAT	E3-UTR	132	96.615	0.97
prcp.S	AGTCTCTACCACCCACTGGA	CGACATCCCACATAAACCCTGTA	E3-E4J	110	95.233	0.994
eef1a1.S	TGGATATGCCCCTGTGTTGGATT	TCCACGCACATTGGCTTTCCT	(Fini et al. 2012; Dubois et al. 2006)			
odc1.S	TGAAAACATGGGTGCCTACA	AAGTTCCATTCCGCTCTCCT				
dio1	CAGCAGATGAATGGGCATTGA	TGTCTAACACTACTGGGCAAGAAGGT				
dio2	CAAATCCAGCCAGAGAGTCAATGG	TGACCTGCTTGTAGGCATCCA				
dio3	CACAAAAAGTGCGACCAAACG	GCCTTGTTGCAGTTTACT				
tr-alpha	CGCCTTGGTCTCTTCGGAT	CCAGAAGTGGGGAATGTTGTGTT				
tr-beta	AAGAGTGGTTGATTTTGCCAAAA	AGGGACATGATCTCCATACAACAG				

Supplementary Table 1: Primers for gene expression (RT-qPCR). See material and methods
